# Biological Activities and ADMET-Related Properties of Novel Set of Cinnamanilides †

**DOI:** 10.3390/molecules25184121

**Published:** 2020-09-09

**Authors:** Jiri Kos, Andrzej Bak, Violetta Kozik, Timotej Jankech, Tomas Strharsky, Aleksandra Swietlicka, Hana Michnova, Jan Hosek, Adam Smolinski, Michal Oravec, Ferdinand Devinsky, Milan Hutta, Josef Jampilek

**Affiliations:** 1 Regional Centre of Advanced Technologies and Materials, Faculty of Science, Palacky University, Slechtitelu 27, 78371 Olomouc, Czech Republic; jiri.kos@upol.cz (J.K.); tomas.strharsky01@upol.cz (T.S.); michnova.hana@gmail.com (H.M.); jan.hosek@upol.cz (J.H.); 2 Department of Chemistry, University of Silesia, Szkolna 9, 40007 Katowice, Poland; violetta.kozik@us.edu.pl (V.K.); aswietlicka@us.edu.pl (A.S.); 3 Department of Analytical Chemistry, Faculty of Natural Sciences, Comenius University, Ilkovicova 6, 84215 Bratislava, Slovakia; timotej.jankech@gmail.com (T.J.); milan.hutta@uniba.sk (M.H.); 4 Central Mining Institute, Pl. Gwarkow 1, 40166 Katowice, Poland; smolin@gig.katowice.pl; 5 Global Change Research Institute CAS, Belidla 986/4a, 60300 Brno, Czech Republic; oravec.m@czechglobe.cz; 6 Faculty of Pharmacy, Comenius University, Odbojarov 10, 83232 Bratislava, Slovakia; devinsky@fpharm.uniba.sk

**Keywords:** cinnamamides, synthesis, antistaphylococcal activity, MTT assay, cytotoxicity, lipophilicity, PCA, IVE-PLS, quantitative structure-property relationships

## Abstract

A series of nineteen novel ring-substituted *N*-arylcinnamanilides was synthesized and characterized. All investigated compounds were tested against *Staphylococcus aureus* as the reference strain, two clinical isolates of methicillin-resistant *S. aureus* (MRSA), and *Mycobacterium tuberculosis*. (2*E*)-*N*-[3-Fluoro-4-(trifluoromethyl)phenyl]-3-phenylprop-2-enamide showed even better activity (minimum inhibitory concentration (MIC) 25.9 and 12.9 µM) against MRSA isolates than the commonly used ampicillin (MIC 45.8 µM). The screening of the cell viability was performed using THP1-Blue™ NF-κB cells and, except for (2*E*)-*N*-(4-bromo-3-chlorophenyl)-3-phenylprop-2-enamide (IC_50_ 6.5 µM), none of the discussed compounds showed any significant cytotoxic effect up to 20 μM. Moreover, all compounds were tested for their anti-inflammatory potential; several compounds attenuated the lipopolysaccharide-induced NF-κB activation and were more potent than the parental cinnamic acid. The lipophilicity values were specified experimentally as well. In addition, in silico approximation of the lipophilicity values was performed employing a set of free/commercial clogP estimators, corrected afterwards by the corresponding pK_a_ calculated at physiological pH and subsequently cross-compared with the experimental parameters. The similarity-driven property space evaluation of structural analogs was carried out using the principal component analysis, Tanimoto metrics, and Kohonen mapping.

## 1. Introduction

Inflammatory diseases of visceral organs, joints, bones, etc. can be based on an infectious or autoimmune basis, but the body always aims to eradicate noxious agents and restore tissue homeostasis [1]. A bacterial infection is often connected with severe inflammatory diseases such as bronchial asthma [2], gingivitis, periodontitis [3], systemic lupus erythematosus [4], and even with cancer [5]. Because the bacterial infection can trigger inflammation, which can cause subsequent damage of surrounding tissue [6], it seems advantageous to follow a multi-target approach in drug design and try to prepare molecules with dual antimicrobial and anti-inflammatory activity. Multi-target drug discovery represents an innovative approach of medicinal chemistry to overcome a crisis in drug design, reflected in the small number of newly approved drugs. This approach is based on the concepts of privileged scaffolds, polypharmacology, and multifactorial diseases [7,8,9,10,11,12]. Thus, multi-target drugs can be designed for the simultaneous treatment of, for example, autoimmune, inflammatory, and invasive diseases. From pharmacoeconomic and patients’ comfort point of view, it seems favorable to treat both a cause and a consequence (bacterial infection and inflammation) simultaneously with one active substance.

Cinnamic acids are one of such privileged multi-target structures that occur naturally in all plants [13,14,15]. Cinnamic acids as well as hydroxy- and phenyl-substituted derivatives of cinnamic acids have been widely investigated due to their significant and varied biological effects including anti-inflammatory, antioxidant, hepatoprotective, antidiabetic, antidepressant/anxiolytic, antifungal, antibacterial, antiviral, and anticancer effects [16,17,18,19,20,21,22,23,24]. Derivatives of cinnamic acids are also used as agricultural fungicides [25]. Ring-substituted *N*-arylcinnamanilides were recently synthesized and tested for their antibacterial, antimycobacterial, anti-inflammatory potential and antifungal activity as well as for their activity related to the inhibition of photosynthetic electron transport (PET) in spinach (*Spinacia oleracea* L.) chloroplasts [26,27,28]. Since the *N*-phenylcinnamamide skeleton can be considered as a privileged scaffold providing multi-target agents, new di-, tri-, and tetra-halogenated *N*-arylcinnamanilides were prepared.

The biopharmaceutical profile of a compound is increasingly relevant for characterizing both the pharmacokinetic (ADMET) and pharmacodynamic aspects of drug–receptor/enzyme interactions [29,30]. The ADMET-friendly design of molecular in vivo permeability and cell bioaccumulation belongs to the field of the quantitative structure-property relationships (QSPR), where the physicochemical properties of a compound are the mathematical function of the chemical composition/constitution [31,32]. In this context, lipophilicity and its quantitative descriptor (logP) that often correlates well with the bioactivity of compounds indicate the differential partitioning of a neutral compound between two immiscible solvents (*n*–octanol/water) under equilibrium conditions [33,34]. The existing experimental lipophilicity dataset is negligible (3 × 10^4^) when compared to the enormous number of compounds under design; therefore, the reliable measure/estimation of lipophilicity is a valid requirement at the early stages of drug design [35]. Hence, an attractive alternative for lipophilicity estimation is in silico predictive protocols due to the additive-constitutive nature of the logP descriptor [36]. Regrettably, the comparative evaluation of implemented computational algorithms (linear or non-linear) revealed that the practical development of magic bullet (a global model for a diverse set of structural types) is dubious—the quality of lipophilicity estimation varies noticeably depending on the chemical type under consideration [37]. In order to eliminate a consequential uncertainty (over-/under-estimation) of clogP value, a possible vast range of in silico predictors should be engaged and compared with the available experimental data. As a matter of fact, early lipophilicity profiling (theoretical and/or empirical) might facilitate better decision-making in the hit→lead→seed→drug route and eradicate bad actors (false positive hits) at early stages of drug design/development according to the “fail fast, fail cheap” concept [38]. Due to the inaccuracy of clogP prediction, the potential exclusion of prospective drug molecules (false negative hits) at the preliminary step of drug discovery might happen using the Ro5 rule [39].

Depending on the target location and the track of administration, chemicals are exposed to pH variations in the physiological environment (pH = 2–12). In the case of non-ionizable compounds, only neutral species exist; however, the majority of commercial pharmaceuticals contain an ionizable moiety—approximately 75% are basic, and 20% are acidic [40]. In fact, even partial ionization of a molecule results in the depletion of calculated/measured lipophilicity; therefore, the distribution coefficient logD_pH_ (the pH dependent version of logP) should be taken into consideration [41]. In fact, logD_pH_ potentially accumulates errors due to the logP and dissociation constant (pK_a_) estimations according to the following formulas: ${\mathrm{logD}}_{\mathrm{pH}}\;=\;\mathrm{logP}-\log\left[1+10^{\left(\mathrm{pH}-{\mathrm{pK}}_{a}\right)}\right]$ for acids and ${\mathrm{logD}}_{\mathrm{pH}}\;=\;\mathrm{logP}-\log\left[1+10^{\left({\mathrm{pK}}_{a}-\mathrm{pH}\right)}\right]$ for bases [42]. On the other hand, in many systems, ionic species enter the non-aqueous phase, and therefore a more rigorous approach would be appropriate, but this is beyond the scope of this paper.

In the presented study, a set of cinnamic acid anilide derivatives was synthesized, investigated for biological activity, and characterized by a series of experimental lipophilicity values generated using reversed-phase high-performance liquid (RP-HPLC) and reversed-phase thin-layer chromatography (RP-TLC). The similarity-related property space evaluation for the congeneric series of structurally-based analogs was carried out using the principal component analysis (PCA). Moreover, the in silico approximation of the lipophilic values for the ensemble of anilides **1**–**20** was performed employing a set of free/commercial clogP estimators, corrected afterwards by the corresponding pK_a_ calculated at physiological pH and subsequently cross-compared with the experimental parameter. The mean values of the selected molecular descriptors that average over the chosen calculation methods were subsequently correlated with the logD_7.4_ parameter, namely consensus clogP. Finally, the similarity-driven investigation using Tanimoto metrics and Kohonen mapping was conducted revealing some structural dissimilarities within the analyzed series of compounds.

## 2. Results and Discussion

### 2.1. Synthesis and Biological Screening

**Scheme 1:**
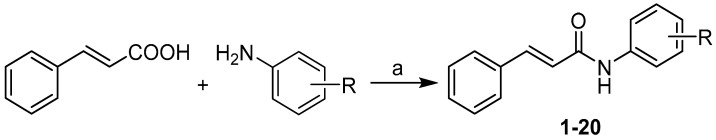
Synthesis of ring-substituted (2*E*)-*N*-aryl-3-phenylprop-2-enamides **1**–**20**. Reagents and conditions: (a) PCl_3_, chlorobenzene, MW (max. 500 W), 130 °C, 30 min [26,28].

All compounds were synthesized from cinnamic acid in a microwave reactor: The carboxyl group was converted with phosphorus trichloride to acyl chloride, which then reacted with the appropriately substituted aniline to give the desired product (Scheme 1). The structures and biological activities are reported in Table 1, and empirically evaluated lipophilicity using RP-HPLC and RP-TLC are listed in Table 2.

#### 2.1.1. In Vitro Antimicrobial Evaluation

The investigated compounds were tested on their antistaphylococcal activity against three clinical isolates of methicillin-resistant *Staphylococcus aureus* (MRSA) and *S. aureus* ATCC 29213 as the reference and quality control strain. Moreover, all compounds were tested against *Mycobacterium tuberculosis* ATCC 25177/H37Ra (see Table 1). As a matter of fact, all compounds showed very limited antimicrobial activity with the exception of compound **10** (R = 3-F-4-CF_3_) that revealed a significant effect against both *S. aureus* and MRSA isolates. It confirmed our previous findings, where compounds with CF_3_ moiety showed high antistaphylococcal and antitubercular activity [26,27]. Consequently, it was expected that other compounds with the CF_3_ motif, such as **13** (R = 2-Br-5-CF_3_) and **19** (R = 2,6-Br-4-CF_3_) or compound **6** (R = 3,4,5-Cl), derived from 3,5-Cl (highly effective against *M. tuberculosis*) should have similar activity [26]. Unfortunately, only molecule **10** turned out to be active. Because the minimum inhibitory concentrations (MICs) of compound **10** are the same against both *S. aureus* and MRSA isolates (25.9 and 12.9 μM), it can be speculated concerning the specific activity against *Staphylococcus* sp. In addition, all compounds were verified against the reference strain *Enterococcus faecalis* ATCC 29212 and three vanA-carrying vancomycin-resistant *E. faecalis* (VRE) isolates; however no activity was recorded.

It is should be emphasized that besides MIC values, minimum bactericidal concentrations (MBCs) were also determined for compound **10**. All MBC values were equal to the MIC values, indicating that compound **10** showed not only bacteriostatic but bactericidal activity. In fact, a tested compound is classified as bactericidal if the ratio of its MBC/MIC values is ≤4 [43].

Finally, 3-(4,5-dimethylthiazol-2-yl)-2,5-diphenyltetrazolium bromide (MTT) assay of effective antistaphylococcal compound **10** was conducted as well. The MTT test is employed to assess cell growth by measuring respiration. The MTT measured viability of bacterial cells less than 70% [44] after the exposure to the MIC values of tested compound is considered as a positive result of this assay, because this low level of cell viability indicates the inhibition of cell growth by the inhibition of respiration [45]. It can be concluded that compound **10** showed a significant decrease in viability to 13.4 ± 0.1%, which is far below the limit of 70% viability of *S. aureus* ATCC 29213 at the tested concentration equal to MIC (i.e., 25.9 µM (8 µg/mL).

#### 2.1.2. In Vitro Cell Viability and Anti-Inflammatory Potential

In vitro cell viability of all the compounds was estimated using the human THP1-Blue™ NF-κB cell line. Almost all tested compounds showed insignificant cytotoxic effect (IC_50_ > 20 µM) (Table 1) with exception of molecule **11** (R = 3-Cl-4-Br, IC_50_ = 6.5 ± 1.0 µM). It seems that the disubstitution of C_(3,4)_’ by highly lipophilic and electron-withdrawing moieties (combination of chlorine and bromine) is critical for the toxic effect of the analyzed series of compounds.

**Figure 1:**
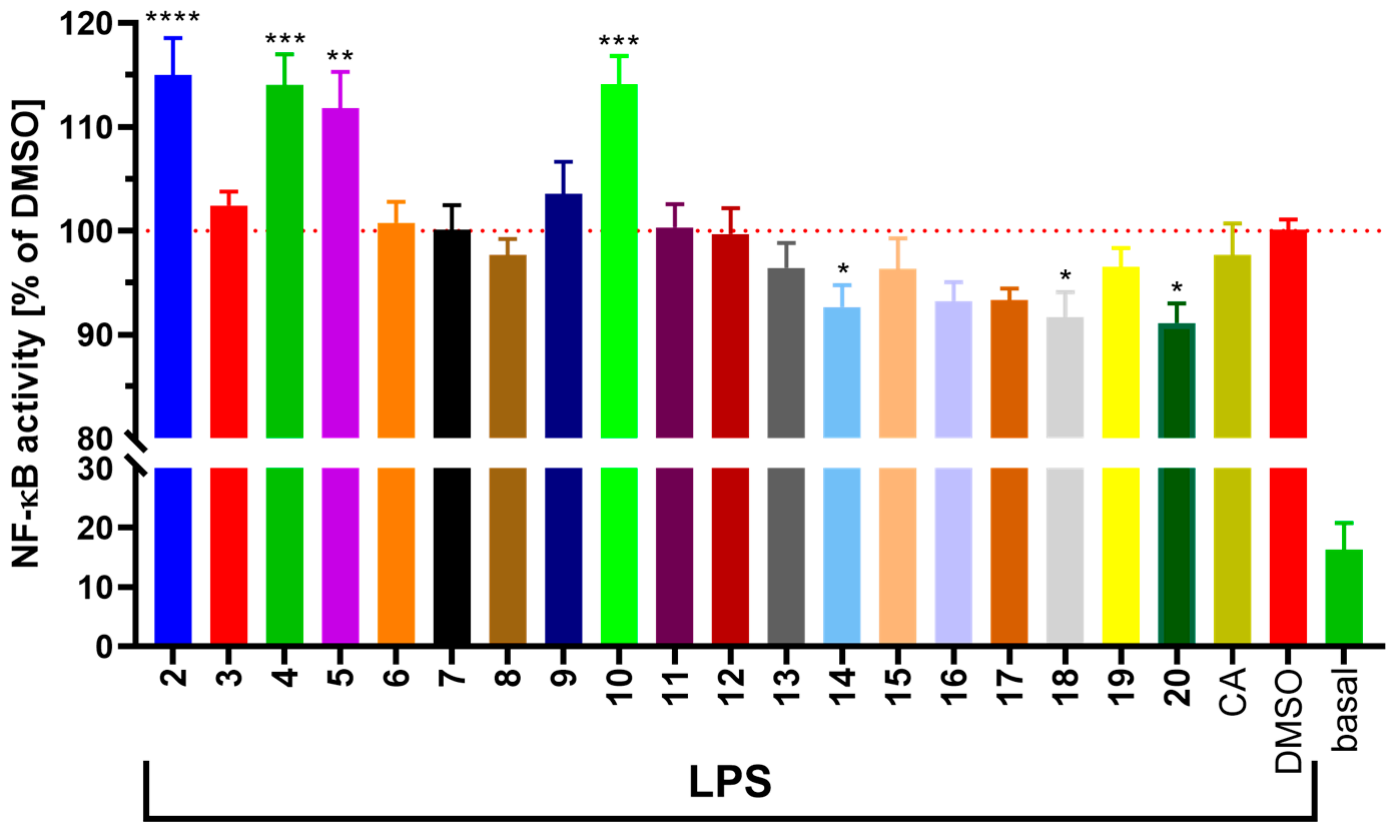
Effect of tested compounds on activity of transcription factor NF-κB. THP1-Blue™ NF-κB cells were pretreated by compounds (1 µM) dissolved in dimethyl sulfoxide (DMSO) for 1 h. Then, lipopolysaccharide (LPS) 1 µg/mL was added to trigger the NF-κB activation. The activity of NF-κB was measured 24 h after LPS stimulation using QuantiBlue™ assay. The graph represents mean ± SEM (n = 9). The red dotted line shows the value of the DMSO-only treated group. Groups were compared with the help of the one-way ANOVA test followed by Fisher’s LSD multiple comparison test. * indicates statistical significance (*p* < 0.05) to DMSO group; ** indicates statistical significance (*p* < 0.01) to DMSO group; *** indicates statistical significance (*p* < 0.001) to DMSO group; **** indicates statistical significance (*p* < 0.0001) to DMSO group. CA—cinnamic acid.

With respect to the used THP1-Blue™ NF-κB cells, the anti-inflammatory potential of compounds was evaluated in order to modulate the activity of the pro-inflammatory transcription nuclear factor (NF)-κB (see Figure 1). After lipopolysaccharide (LPS) stimulation, compounds **14** (R = 2-CF_3_-4-F), **18** (R = 3-CF_3_-4-NO_2_), and **20** (R = 2,6-Br-3-Cl-4-F) showed the highest attenuation of the activity of this transcription factor within the studied set of compounds; however, the decrease was approximately 9%, which was lower compared to the previously verified cinnamides [28]. On the other hand, the half concentration of tested compounds was used in the study. As a matter of fact, compounds **2** (R = 2,4,6-F), **4** (R = 2,4-Cl), **5** (R = 2,4,5-Cl), and **10** (R = 3-F-4-CF_3_) significantly increased the activity of NF-κB by 10–15%, respectively. It seems that the position and type of substituents on phenyl ring is important for tuning pro-/anti-inflammatory potential of cinnamic acid anilides, which confirms similar observations in our previous study, where the substitution of *ortho* and *meta* positions of the anilide ring by rather lipophilic and bulky moieties was preferred for the anti-inflammatory potential [28].

Surprisingly, the insignificant anti-inflammatory potential of compound **13** (R = 2-Br-5-CF_3_) is disappointing, because its chlorinated analogue showed promising activity [28]. It can be hypothesized that the investigated *N*-arylcinnamanilides may have a different mode of action, which may be the nuclear translocation of NF-κB inhibition, or affecting its binding to DNA, or acting by epigenetic regulation, while combinations of these effects are not excluded.

### 2.2. Similarity-Driven Property Evaluation

**Figure 2:**
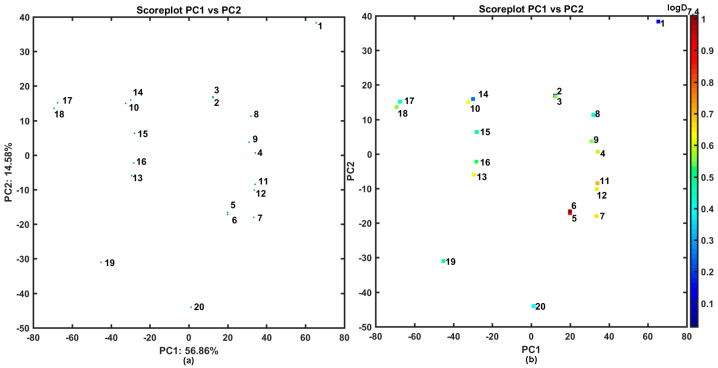
Projection of cinnamic acid anilides **1**–**20** on plane defined by first vs. second principal components for Dragon descriptors (**a**) with experimental logD_7.4_ (**b**). Colors code numerical values of logD at pH = 7.4.

The similarity-guided assessment of the property profile for the group of structurally alike compounds was conducted for the congeneric set of cinnamic acid anilide analogues. The a priori calculation of molecular descriptors, regarded as the result of mathematical transformation of chemical information encoded within a symbolic representation of a molecule is crucial for the compound’s bioavailability and hence critical for the prospective drug candidate properties. The gold standard of ADMET–tailored property approximation is the generation of statistically robust models, where a property is a function of the chemical structure, that are capable of making accurate quantitative predictions, including those of molecular binding affinity, metabolic/pharmacokinetic/ pharmacodynamics fate, or environmental ecotoxicology. The molecular descriptors are essentially estimated based on the molecular structure as intuitive roadmaps even before the synthesis of the molecule has been rationalized. In fact, the majority of topological descriptors is highly intercorrelated; therefore, it is necessary to employ the linear (e.g., PCA) or non-linear (e.g., neural network) techniques to reduce the data dimensionality and illustrate the object similarity in the orthogonal basis using the pair-wise descriptor-based structural resemblance/relatedness measure of the intermolecular resemblance between two objects (e.g., Euclidean metric) [46]. Initially, the principal component analysis (PCA) was performed on the pool of 2567 descriptors calculated using Dragon 6.0 program, where constant and nearly constant variables (standard deviation <10^−4^) were a priori eradicated. Subsequently, the multidimensional matrix was generated (X_20×2567_) with rows and columns representing variables (descriptors) and objects (molecules), respectively. The PCA procedure was employed on the centered and standardized dataset. The compression efficacy of the PCA method is strictly related to the number of uncorrelated variables; therefore, the high percentage of total variance explained by the first three principal components (PC1 + PC2 + PC3 = 77.98%) implies, that descriptors are highly inter-correlated. In fact, first two PCs described 71.44% of the total variance; hence, the chosen properties were projected on the plane specified by the PC1 versus PC2-predicted scores as illustrated in Figure 2a.

Basically, two subfamilies can be formed along the positive and negative values of PC1 for the analyzed **1**–**20** anilides that share a common chemotype based on styrene-containing motif, peptide-like linker, and phenyl R-substituted ring. The first group (PC1 > 0) contains di/tri/tetra-halogenated analogues (objects **2**–**9**, **11**, **12**, **20**), while the second one (PC1 < 0) is composed of mono-trifluoromethyl-based molecules (objects **10**, **13**–**19**). Interestingly, compounds **19** and **20** are located distinctly along the second principal component (PC2 < −30). Not surprisingly, unsubstituted (only hydrogenated) molecule **1** is separated from the remaining ones. The similarity-driven analysis of the empirical lipophilicity at the physiological pH = 7.4 is illustrated on the PC1 vs. PC2 plane. In fact, the most lipophilic molecules **5**–**7** and **11** are grouped together as shown in Figure 2b.

**Figure 3:**
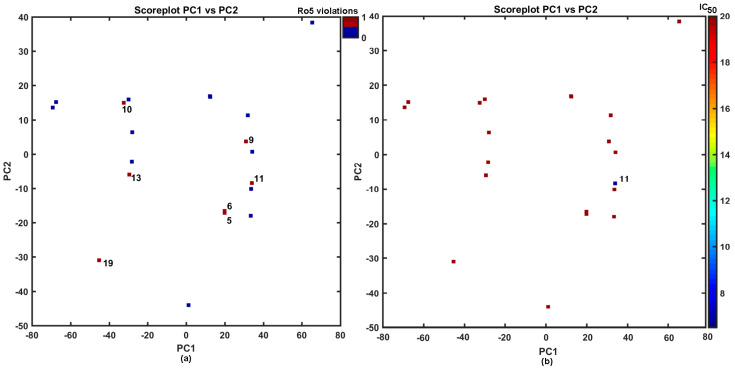
Projection of cinnamic acid anilides **1**–**20** on a plane defined by first vs. second principal components for Dragon descriptors with Ro5 rule violations (**a**) and cytotoxic effect IC_50_ (**b**). Colors code numerical values of Ro5 violations and IC_50_.

On the whole, the vast number of the investigated anilides abide the Lipinski’s Rule of Five (Ro5), where the ADMET-friendly properties are confined by the liminal values imposed on the specific molecular descriptors (MW ≤ 500, HBD ≤ 5, HBA ≤ 10, clogP ≤ 5). Interestingly, the Ro5 violation observed for molecules depicted in Figure 3a is strictly related with the calculated lipophilicity values (clogP > 5). Despite the drug-like property space is still a questionable concept, because a good drug-like score does not make a molecule a drug and vice versa, the Ro5 rule might be handy in differentiating a prospective drug from a non-drug molecule [47]. Moreover, almost all tested compounds showed very low cytotoxic effect (IC_50_ > 20 µM) as presented in Figure 3b and Table 1. Compound **11** (IC_50_ = 6.5 µM) is the visible exception, which is also annotated with Ro5 violation. As mentioned above, it seems that the C_(3,4)_’ disubstitution by chlorine and bromine is critical for the viability of cells unlike the disubstitution of C_(2,4)_’ positions. Since the lipophilicity is regarded as a meaningful property in the context of pharmacokinetic and pharmacodynamic drug-receptor interactions, the lipophilic profiles of the analyzed anilide analogues were thoroughly investigated.

**Figure 4:**
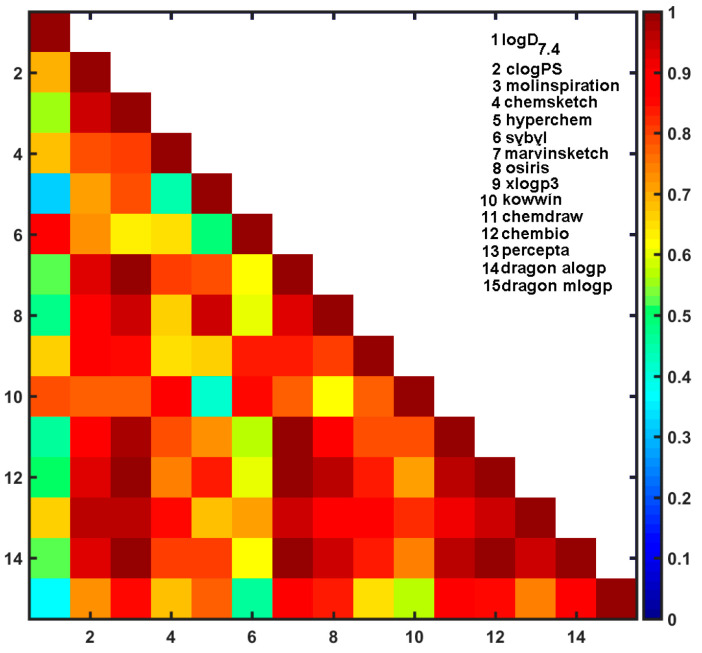
Matrix of correlation coefficients of linear relationships between experimental and calculated logD_7.4_ lipophilicity specified using different programs.

From the drug hunter perspective, the justifiable precariousness of the validity of lipophilicity estimation can arise, using a variety of theoretical approaches, because some methods for the theoretical calculation of lipophilicity might be more or less suitable for specific/heterogeneous series of compounds. In other words, the non-trivial question arises how to single out the best-suited method or combination of methods for clogP estimation of new compounds. It is known that a jack of all trades is a master of none; therefore, it seems advisable not to rely solely on one clogP estimator, but rather a combination of methods should be engaged with subsequent comparison of the results with the experimental data. Hence, a consensus procedure for clogP prediction was previously proposed based on a consensus methodology that was adopted from the structure-based studies, where many docking programs and scoring functions can be employed [48,49,50]. Thus, in silico approximation of the lipophilic values for the ensemble of anilides **1**–**20** was performed employing a set of free/commercial clogP estimators such as clogPS, Molinspirations, OSIRIS, HyperChem 7.0, Sybyl-X, MarvinSketch 15, ACD/ChemSketch 2015, Dragon 6.0, Kowwin, XlogP3, ChemDraw, and ACD/Percepta (Table S1 in Supplementary Materials). Moreover, the deduced clogP values were corrected afterwards by the corresponding pK_a_ calculated at specific pH using ACD/Percepta/pKa Classic module (Table S2 in Supplementary Materials). The obtained clogD_pH_ does not differ significantly from clogP specified by distinct in silico principles, because the investigated molecules do not contain an ionizable moiety. Furthermore, the clogD_7.4_ findings were inter-correlated with each other and cross-compared with the empirical logD_7.4_ values as illustrated by the triangular matrix of linear correlations parameters in Figure 4.

In fact, relatively good correlation (r > 0.65) between experimental logD_7.4_ and model-predicted clogD_7.4_ values was recorded for clogPS, ChemSketch, Sybyl, XlogP3, and Kowwin estimators. On the other hand, rather poor correlation (r ≤ 0.4) was revealed for data provided by HyperChem and Dragon predictors (see Figure 4). The noticeable variations in logD estimation at given pH probably resulted from different computational algorithms (atom/fragment- or descriptor-based) implemented in the software and/or training data engaged at the training stage. Obviously, prediction of the individual lipophilic contribution of each group/atom (sometimes augmented by the structural correction factors) is as good as the modeling data used at the training stage of model generation [51]. In other words, the poorer performance of some predictors focuses on issues arising from the lipophilicity estimation for the in house collection of molecules with structural features uncovered by chemical classes of compounds in the training subset—a wide range of chemical space is still not covered [52]. Luckily, usually a few empirically measured values are sufficient to produce reliable lipophilicity estimation for structurally related series of molecules. Hence, the integrated clogD_7.4_ matrix (X_20×14_) and empirical logD_7.4_ were subjected to the backward elimination PLS-based procedure (IVE) indicating Sybyl-X, XlogP3, Kowwin, ACD/Percepta, and HyperChem as valid contributors to the final QSPR model ($q_{cv}^{2}\;=\;0.72,\;q_{test}^{2}\;=\;0.9,\;A_{opt}\;=\;7$). The mean and median values of the selected estimators that averaged over the chosen clogD_7.4_ values were subsequently correlated with the experimental parameter with correlation coefficient 0.65, because not only the best inter-correlated clogD_7.4_ values were specified in the consensus clogP approach.

**Figure 5:**
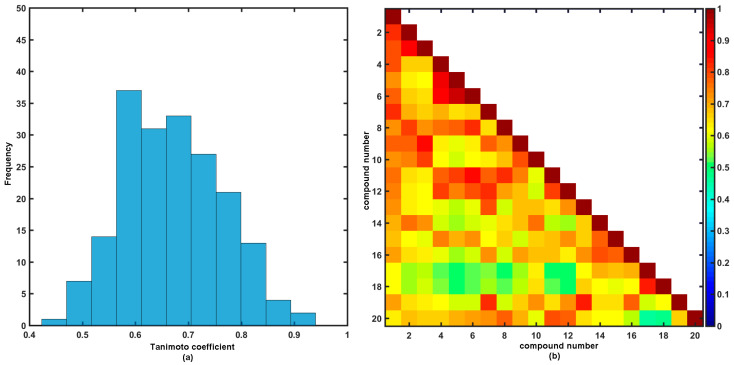
Distribution of Tanimoto coefficients (**a**) and triangular matrix of Tanimoto coefficients (**b**) for **1**–**20** anilides.

The similarity tenet in the chemical space (CS) is the core of many SAR-driven procedures, where structurally alike molecules are expected to display similar physicochemical and/or pharmacological properties [53]. Conceptually, a numerical measure of molecular diversity between two objects can be quantitatively expressed by a bit-string representation (sometimes augmented with the scaling coefficients) in the function of (un-)common features. Unarguably, it is still a powerful concept despite some obvious oversimplification of the similarity quantification (e.g., some similarity scores exhibit size-dependent behavior) [54]. The pairwise relatedness between descriptor-guided structures can be numerically evaluated by a variety of relative distance metrics (e.g., Hamming or Euclidean measures) or absolute comparison using Tanimoto coefficient calculated for molecular fingerprints (e.g., OpenBabel) [55]. The distribution of Tanimoto coefficients for the ensemble of investigated anilides **1**–**20** is illustrated in Figure 5a, where the highest frequency was recorded in the pretty wide range of 0.55 < T < 0.75. The detailed inspection of the symmetric Tanimoto coefficient matrix (T_20×20_) in Figure 5b reveals the structural dissimilarities of nitro-substituted isomers (compounds **17** and **18**) as compared with the remaining ones that confirms our previous PCA (PC1 < −60) findings (see Figure 2a). Interestingly, mono-bromo/chloro- substituted isomers (molecules **11** and **12**) indicate the structural similarity to di-/tri-bromo/chloro- substituted positional isomers (compounds **4**–**9**) as was also shown in Figure 2a (PC1 > 20). Unfortunately, the similarity investigation did not provide valuable hints that could explain the noticeable variations in the toxic effect exerted by molecule **11** (see Figure 3b) and the remaining ones.

#### Comparative Molecular Shape Analysis

**Figure 6:**
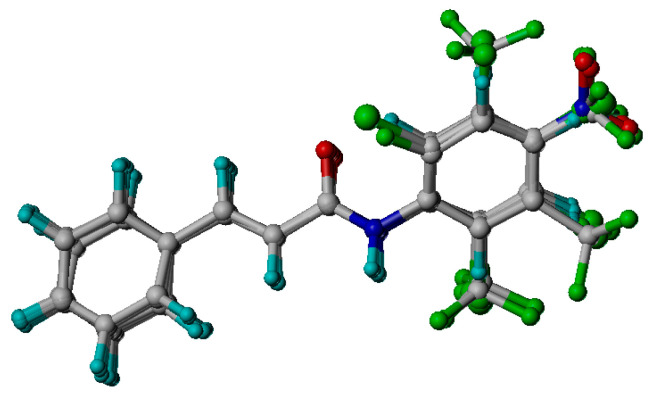
Superimposition of **1**–**20** anilides according to AAA hypothesis.

The molecular shape analysis of the cinnamic acid anilides was performed, because the molecular electrostatics and lipophilicity are two important properties used in the rational drug design. Firstly, the 11-ordered atom trial alignment of the most active molecule **10** (active analogue approach, AAA) was applied to cover the entire bonding topology in the maximal common structure (MCS) in the FIT method as illustrated in Figure 6.

**Figure 7:**
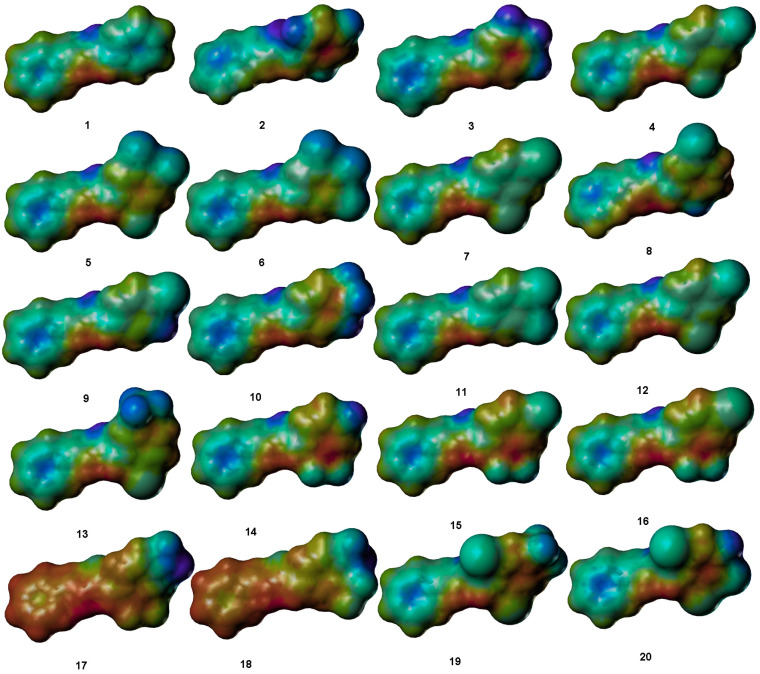
Molecular electrostatic potential topography of **1**–**20** anilides. Blue color codes negative values; red color codes positive MESP values.

Secondly, the molecular electrostatic potentials (MESP) on the Connolly surface were specified to provide information on the charge distribution of the substituted anilides. The interrelation between electrostatic and lipophilicity potentials on molecular surfaces was reported previously [56]. The electron-rich positions seem to appear at low, possibly negative, electrostatic potential energy values (see Figure 7). In other words, low MESP values indicate molecular areas susceptible to electrophilic attack (nucleophilic positions), while greater electrostatic values correspond to electrophilic positions. Noticeably, the negative MESP values (dark blue areas) in the close proximity of the substituted anilide ring seem to contribute to the MIC activity of molecule **10**. Obviously, the introduction and composition of electronegative or electropositive substituents have an impact on the lipophilicity values as well.

**Figure 8:**
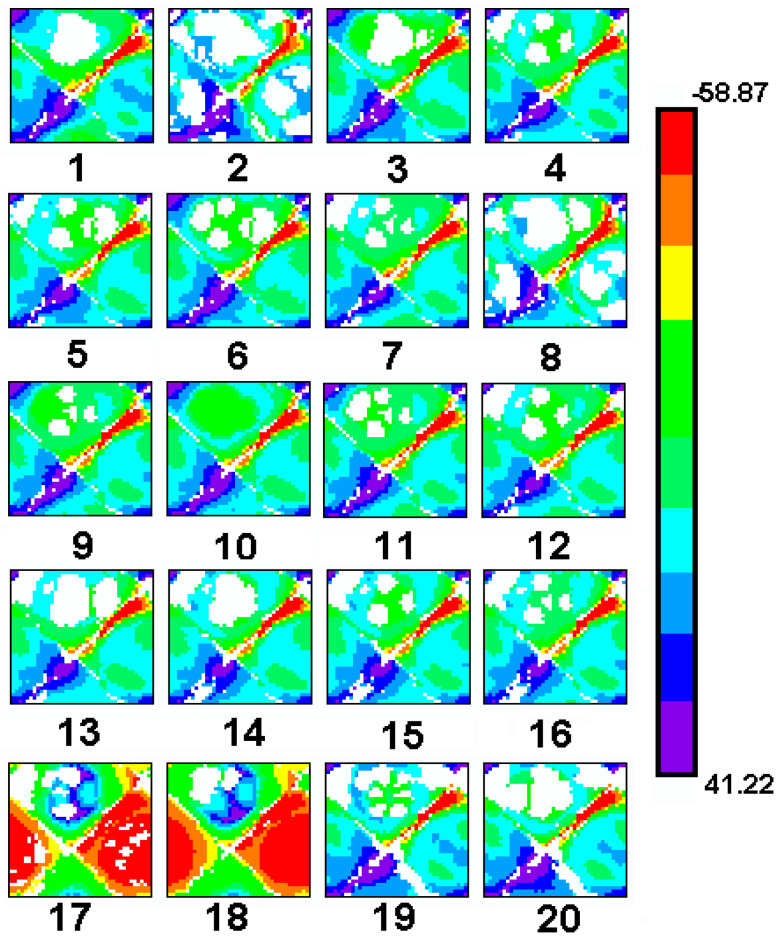
Comparative SOM 40 × 40 maps for **1**–**20** anilides. Colors code MESP values, white color indicates non-active neurons.

The spatial MESP distribution does not provide a practical tool for molecular comparison; therefore, simpler, comparative 2D Kohonen maps of the entire molecular surface were generated [57]. Self-organizing Kohonen neural mapping (SOM) is a nonlinear projection procedure that reduces the input data dimensionality (e.g., converts 3D objects to 2D), while preserving the topological relationships between the input and output data [58]. Moreover, a trained network can be engaged to project the specified molecular property (expressed as a vector) by generating a 2D color-coded clustering pattern called a feature map. The SOM algorithm was employed to generate an electrostatic potential map in the form of a two-dimensional topographic pattern produced from input signals (points) that were sampled randomly at the molecular surface as illustrated in Figure 8. The substitution of the anilide ring by a rather bulky motif (e.g.,-CF_3_) was preferred for the anti-inflammatory potential and lipophilic compound property; therefore, a mono-trifluoro- methyl-based molecule with highly electronegative substituent (e.g.,-F) was chosen as a template (the most active molecule **10**) to proceed the remaining (counter-template) molecules. The structural inconsistences (dissimilarities) within the analyzed set of compounds are indicated by the number of inactive (empty/white) neurons in the map (see Figure 8). Unfortunately, there are no clear and visible variations in the surface charge distribution within 40 × 40 maps between substituted analogues, with exception of molecules **17** and **18**. Not surprisingly, the lowest number of non-active neurons of the comparative maps (less than 200 out of 1600) is prescribed to the similarly substituted (position 3 and 4 of the anilide ring) molecules **9**, **11,** and **18**.

## 3. Materials and Methods

### 3.1. Chemistry

#### 3.1.1. General Information

All reagents were purchased from Merck (Sigma-Aldrich, St. Louis, MO, USA) or Alfa (Alfa-Aesar, Ward Hill, MA, USA). Reactions were performed using a CEM Discover SP microwave reactor (CEM, Matthews, NC, USA). Melting points were determined on an apparatus Stuart SMP10 (Stone, UK) and are uncorrected. Infrared (IR) spectra were recorded on an ATR Zn/Se for a Nicolet™ iS 5 FT-IR spectrometer (Thermo Fisher Scientific, West Palm Beach, FL, USA). The spectra were obtained by the accumulation of 64 scans with 4 cm^−1^ resolution in the region of 4000–400 cm^−1^. All ^1^H- and ^13^C-NMR spectra were recorded on a JEOL JNM-ECA 600II NMR spectrometer (600 MHz for ^1^H and 150 MHz for ^13^C, Jeol, Tokyo, Japan) in dimethyl sulfoxide-*d_6_* (DMSO-*d*_6_); ^1^H and ^13^C chemical shifts (δ) are reported in ppm. High-resolution mass spectra were measured using a high-performance liquid chromatograph Dionex UltiMate^®^ 3000 (Thermo Scientific, West Palm Beach, FL, USA) coupled with an LTQ Orbitrap XL^TM^ Hybrid Ion Trap-Orbitrap Fourier Transform Mass Spectrometer (Thermo Scientific) equipped with a HESI II (heated electrospray ionization) source in the negative mode.

#### 3.1.2. Synthesis

Cinnamic acid (3.37 mM) was suspended at room temperature in dry chlorobenzene (20 mL) inside a microwave tube, where phosphorus trichloride (1.7 mM) and the corresponding aniline derivative (3.37 mM) were added dropwise. Following this step, a magnetic stirrer was added to the tube and the reaction mixture was transferred to the microwave reactor (max. 500 W) at 130 °C for 30 min, where the synthesis at elevated pressure was performed. After the mixture was cooled to 60 °C, the solvent was evaporated in vacuum. A solid residue was washed with 2M HCl, and a crude product was recrystallized, using 96% ethanol first, and then 50% ethanol [26,28].

(2E)-*N*-Phenyl-3-phenylprop-2-enamide (**1**) was described previously by Pospisilova et al. [26].

*(2E)-3-Phenyl-N-(2,4,6-trifluorophenyl)prop-2-enamide* (**2**)*,* Yield 72%; Mp 134–136 °C; IR (cm^−1^): 3244, 3078, 3024, 1659, 1631, 1612, 1525, 1443, 1336, 1239, 1177, 1124, 1045, 999, 972, 861, 843, 764, 751, 705, 666, 612, 572, 533, 513, 481; ^1^H-NMR (DMSO-*d_6_*), δ: 9.91 (s, 1H), 7.65–7.64 (m, 2H), 7.61 (d, *J* = 15.8 Hz, 1H), 7.47–7.41 (m, 3H), 7.32 (t, *J* = 8.2 Hz, 2H), 6.85 (d, *J* = 15.8 Hz, 1H); ^13^C-NMR (DMSO-*d_6_*), δ: 163.95, 159.93 (d, *J* = 245.7 Hz), 157.88 (ddd, *J* = 250.0 Hz, 15.9 Hz, 8.7 Hz), 141.19, 134.44, 130.02, 129.03, 127.87, 120.35, 111.48 (td, *J* = 17.3 Hz, *J* = 5.8 Hz), 100.95 (m) (Figure S1); HR-MS: C_15_H_9_ONF_3_ [M − H]^−^ calculated 276.0642 *m/z*, found 276.0634 *m/z* (Figure S2).

*(2E)-3-Phenyl-N-(3,4,5-trifluorophenyl)prop-2-enamide* (**3**), Yield 74%; Mp 169–171 °C; IR (cm^−1^): 3303, 1661, 1617, 1545, 1530, 1451, 1426, 1338, 1238, 1208, 1195, 1044, 1013, 976, 858, 848, 805, 770, 761, 717, 642, 628, 561, 509, 481; ^1^H-NMR (DMSO-*d_6_*), δ: 10.58 (s, 1H), 7.64–7.67 (m, 5H), 7.46–7.41 (m, 3H), 6.74 (d, *J* = 15.8 Hz, 1H); ^13^C-NMR (DMSO-*d_6_*), δ: 164.02, 150.09 (ddd, *J* = 244.2 Hz, *J* = 10.1 Hz, *J* = 5.8 Hz), 141.33, 135.56 (td, *J* = 11.6 Hz, *J* = 4.3 Hz), 134.70 (dt, *J* = 244.2 Hz, *J* = 15.9 Hz), 134.38, 130.10, 129.05, 127.89, 121.25, 103.45 (m) (Figure S3); HR-MS: C_15_H_9_ONF_3_ [M − H]^−^ calculated 276.0642 *m/z*, found 276.0633 *m/z* (Figure S4).

*(2E)-N-(2,4-Dichlorophenyl)-3-phenylprop-2-enamide* (**4**), Yield: 62%; Mp 159–161 °C; IR (cm^−1^): 3264, 3071, 3027, 1654, 1620, 1578, 1524, 1471, 1448, 1380, 1335, 1283, 1238, 1202, 1182, 1144, 1099, 1072, 1053, 1029, 1005, 999, 969, 858, 826, 787, 757, 725, 710, 697, 658, 632, 560, 559, 513, 488, 445; ^1^H-NMR (DMSO-*d_6_*), δ: 9.78 (s, 1H), 7.98 (d, *J* = 8.9 Hz, 1 H), 7.69 (d, *J* = 2.1 Hz, 1H), 7.65–7.64 (m, 2H), 7.62, (d, *J* = 15.8 Hz, 1H), 7.47–7.41 (m, 4H), 7.11 (d, *J* = 15.8 Hz, 1H); ^13^C-NMR (DMSO-*d_6_*), δ: 164.02, 141.17, 134.62, 134.21, 129.99, 129.09, 129.05, 128.95, 127.88, 127.59, 126.57, 126.43, 121.54 (Figure S5); HR-MS: C_15_H_10_ONCl_2_ [M − H]^−^ calculated 290.01450 *m/z*, found 290.0140 *m/z* (Figure S6).

*(2E)-3-Phenyl-N-(2,4,5-trichlorophenyl)prop-2-enamide* (**5**), Yield: 52%; Mp 170–172 °C; IR (cm^−1^): 3265, 3107, 3061, 3011, 1656, 1629, 1600, 1568, 1511, 1456, 1446, 1364, 1281, 1248, 1202, 1181, 1129, 1074, 1031, 962, 942, 880, 855, 798, 756, 729, 706, 688, 675, 631, 579, 564, 499, 465, 448; ^1^H-NMR (DMSO-*d_6_*), δ: 9.85 (s, 1H), 8.33 (s, 1H), 7.94 (s, 1H), 7.66–7.65 (m, 2H), 7.64 (d, *J* = 15.8 Hz, 1H), 7.48–7.42 (m, 3H), 7.16 (d, *J* = 15.8 Hz, 1H).; ^13^C-NMR (DMSO-*d_6_*), δ: 164.23, 141.68, 135.18, 134.52, 130.55, 130.12, 129.84, 129.05, 127.95, 126.89, 125.31, 124.45, 121.29 (Figure S7); HR-MS: C_15_H_9_ONCl_3_ [M − H]^−^ calculated 323.9755 *m/z*, found 323.9751 *m/z* (Figure S8).

*(2E)-3-Phenyl-N-(3,4,5-trichlorophenyl)prop-2-enamide* (**6**), Yield: 75%; Mp 237–239 °C; IR (cm^−1^): 3157, 3080, 1655, 1613, 1583, 1513, 1433, 1378, 1337, 1281, 1245, 1196, 1188, 1148, 1011, 998, 967, 944, 880, 860, 815, 762, 711, 685, 617, 602, 575, 536, 483; ^1^H-NMR (DMSO-*d_6_*), δ: 10.62 (s, 1H), 7.95 (s, 2H), 7.65–7.62 (m, 3H), 7.47–7.42 (m, 3H), 6.74 (d, *J* = 15.8 Hz, 1H); ^13^C-NMR (DMSO-*d_6_*), δ: 164.16, 141.63, 139.28, 134.31, 132.91, 130.20, 129.08, 127.96, 123.40, 121.14, 119.19 (Figure S9); HR-MS: C_15_H_9_ONCl_3_ [M − H]^−^ calculated 323.9755 *m/z*, found 323.9752 *m/z* (Figure S10).

*(2E)-N-(2,4-Dibromophenyl)-3-phenylprop-2-enamide* (**7**), Yield: 49%; Mp 180–182 °C; IR (cm^−1^): 3263, 2980, 2888, 1653, 1620, 1575, 1521, 1464, 1446, 1376, 1336, 1280, 1240, 1203, 1184, 1080, 1040, 1007, 967, 859, 825, 766, 755, 711, 688, 643, 619, 566, 546, 501, 472, 440; ^1^H-NMR (DMSO-*d_6_*), δ: 9.68 (s, 1H), 7.94 (d, *J* = 2.1 Hz, 1H), 7.78 (d, *J* = 8.9 Hz, 1H), 7.65–7.60 (m, 4H), 7.47–7.41 (m, 3H), 7.06 (d, *J* = 15.8 Hz, 1H); ^13^C-NMR (DMSO-*d_6_*), δ: 163.93, 141.13, 135.88, 134.61, 134.55, 130.98, 129.99, 129.05, 127.88, 127.73, 121.50, 117.97, 117.66 (Figure S11); HR-MS: C_15_H_10_ONBr_2_ [M − H]^−^ calculated 377.9134 *m/z*, found 377.9106 *m/z* (Figure S12).

*(2E)-N-(5-Chloro-2-fluorophenyl)-3-phenylprop-2-enamide* (**8**), Yield: 59%; Mp 118–120 °C; IR (cm^−1^): 3244, 3188, 3119, 3042, 1663, 1622, 1612, 1541, 1481, 1415, 1343, 1268, 1253, 1202, 1182, 1112, 996, 915, 871, 808, 761, 737, 679, 646, 574, 566, 502, 485, 454; ^1^H-NMR (DMSO-*d_6_*), δ: 10.13 (s, 1H), 8.31 (dd, *J* = 6.9 Hz, *J* = 2.7 Hz, 1H), 7.64–7.61 (m, 3H), 7.47–7.41 (m, 3H), 7.35 (dd, *J* = 11.0 Hz, *J* = 8.9 Hz, 1H), 7.22–7.19 (m, 1H), 7.11 (d, *J* = 15.8 Hz, 1H); ^13^C-NMR (DMSO-*d_6_*), δ: 164.22, 151.55 (d, *J* = 245.6 Hz), 141.31, 134.58, 130.04, 129.06, 128.02 (d, *J* = 2.9 Hz), 127.89 (d, *J* = 13.0 Hz), 127.87, 124.14 (d, *J* = 7.2 Hz), 122.23, 121.48, 116.98 (d, *J* = 21.7 Hz) (Figure S13); HR-MS: C_15_H_10_ONClF [M − H]^−^ calculated 274.0440 *m/z*, found 274.0435 *m/z* (Figure S14).

*(2E)-N-(4-Bromo-3-fluorophenyl)-3-phenylprop-2-enamide* (**9**), Yield: 69%; Mp 146–148 °C; IR (cm^−1^): 3405, 1673, 1625, 1598, 1514, 1473, 1450, 1414, 1333, 1302, 1240, 1204, 1190, 1155, 1135, 1045, 988, 976, 944, 868, 857, 812, 780, 763, 738, 707, 683, 642, 572, 564, 544, 518, 518; ^1^H-NMR (DMSO-*d_6_*), δ: 10.55 (s, 1H), 7.89 (dd, *J* = 8.9 Hz, *J* = 4.0 Hz, 1H), 7.67–7.61 (m, 4H), 7.47–7.41 (m, 3 H), 7.36 (dd, *J* = 8.9 Hz, *J* = 2.1 Hz, 1H), 6.79 (d, *J* = 15.8 Hz, 1H); ^13^C-NMR (DMSO-*d_6_*), δ: 163.95, 158.08 (d, *J* = 242.8 Hz), 141.13, 140.43 (d, *J* = 10.1 Hz), 134.48, 133.41, 130.06, 129.08, 127.88, 121.54, 116.59 (d, *J* = 2.9 Hz), 107.19 (d, *J* = 26.0 Hz), 100.83 (d, *J* = 21.7 Hz) (Figure S15); HR-MS: C_15_H_10_ONBrF [M − H]^−^ calculated 317.9935 *m/z*, found 317.9929 *m/z* (Figure S16).

*(2E)-N-[3-Fluoro-4-(trifluoromethyl)phenyl]-3-phenylprop-2-enamide* (**10**), Yield 69%; Mp 139–141 °C; IR (cm^−1^): 8336, 1671, 1624, 1560, 1521, 1506, 1452, 1424, 1413, 1338, 1319, 1205, 1192, 1162, 1117, 1049, 996, 970, 666, 827, 767, 763, 738, 710, 633, 603, 565, 536, 507, 484; ^1^H-NMR (DMSO-*d_6_*), δ: 10.78 (s, 1H), 7.95 (dd, *J* = 13.7 Hz, 1H), 7.75 (t, *J* = 8.6 Hz, 1H), 7.67 (d, *J* = 15.8 Hz, 1H), 7.68–7.65 (m, 2H), 7.54 (d, *J* = 8.2 Hz, 1H), 7.48–7.42 (m, 3H), 6.82 (d, *J* = 15.8 Hz, 1H); ^13^C-NMR (DMSO-*d_6_*), δ: 164.33, 159.20 (dq, *J* = 248.5 Hz, *J* = 2.9 Hz), 144.96 (d, *J* = 11.6 Hz), 141.74, 134.36, 130.20, 129.08, 127.95, 127.87 (m), 122.82 (q, *J* = 271.7 Hz), 121.25, 114.75, 110.57 (qd, *J* = 31.8 Hz, *J* = 11.6 Hz), 106.65 (d, *J* = 26.1 Hz) (Figure S17); HR-MS: C_16_H_10_ONF_4_ [M − H]^−^ calculated 308.0704 *m/z*, found 308.0695 *m/z* (Figure S18).

*(2E)-N-(4-Bromo-3-chlorophenyl)-3-phenylprop-2-enamide* (**11**), Yield: 61%; Mp 170–172 °C; IR (cm^−1^): 3282, 3097, 2980, 2888, 1663, 1627, 1579, 1521, 1470, 1449, 1376, 1338, 1288, 1253, 1229, 1180, 1113, 1071, 968, 882, 861, 808, 761, 711, 679, 676, 577, 561, 496, 482; ^1^H-NMR (DMSO-*d_6_*), δ: 10.51 (s, 1H), 8.11 (d, *J* = 2.1 Hz, 1H), 7.72 (d, *J* = 8.2 Hz, 1H), 7.65–7.61 (m, 3H), 7.50 (dd, *J* = 8.6 Hz, *J* = 2.4 Hz, 1H), 7.47–7.41 (m, 3H), 6.78 (d, *J* = 15.8 Hz, 1H); ^13^C-NMR (DMSO-*d_6_*), δ: 163.94, 141.13, 139.92, 134.47, 133.95, 133.09, 130.07, 129.08, 127.88, 121.53, 120.31, 119.45, 114.48 (Figure S19); HR-MS: C_15_H_10_ONBrCl [M − H]^−^ calculated 333.9640 *m/z*, found 333.9635 *m/z* (Figure S20).

*(2E)-N-(2-Bromo-4-chlorophenyl)-3-phenylprop-2-enamide* (**12**), Yield: 68%; Mp 173–175 °C; IR (cm^−1^): 3258, 2980, 2888, 1653, 1621, 1572, 1524, 1465, 1447, 1380, 1336, 1279, 1263, 1238, 1201, 1180, 1093, 1039, 1006, 999, 969, 858, 824, 775, 757, 714, 700, 653, 632, 568, 551, 512; ^1^H-NMR (DMSO-*d_6_*), δ: 9.69 (s, 1H), 7.84–7.82 (m, 2H), 7.65–7.64 (m, 2H), 7.62 (d, *J* = 15.8 Hz, 1H), 7.49 (dd, *J* = 8.6 Hz, *J* = 2.4 Hz, 1H), 7.47–7.41 (m, 3H), 7.07 (d, *J* = 15.8 Hz, 1H); ^13^C-NMR (DMSO-*d_6_*), δ: 163.94, 141.08, 135.50, 134.60, 131.87, 129.96, 129.72, 129.02, 128.05, 127.85, 127.38, 121.49, 117.69 (Figure S21); HR-MS: C_15_H_10_ONBrCl [M − H]^−^ calculated 333.9640 *m/z*, found 333.9635 *m/z* (Figure S22).

*(2E)-N-[2-Bromo-5-(trifluoromethyl)phenyl]-3-phenylprop-2-enamide* (**13**), Yield 59%; Mp 134–135 °C; IR (cm^−1^): 3270, 1658, 1631, 1606, 1529, 1467, 1423, 1329, 1262, 1170, 1112, 1078, 1035, 963, 928, 894, 856, 816, 759, 709, 685, 643, 615, 560, 491, 454; ^1^H-NMR (DMSO-*d_6_*), δ: 9.84 (s, 1H), 8.26 (d, *J* = 1.4 Hz, 1H), 7.94 (d, *J* = 8.2 Hz, 1H), 7.67–7.65 (m, 2H), 7.65 (d, *J* = 15.8 Hz, 1H), 7.49–7.42 (m, 4H), 7.14 (d, *J* = 15.8 Hz, 1H); ^13^C-NMR (DMSO-*d_6_*), δ: 164.25, 141.54, 137.26, 134.54, 134.06, 130.08, 129.06, 128.59 (q, *J* = 31.8 Hz), 127.94, 123.71 (q, *J* = 273.1 Hz), 122.77 (q, *J* = 4.3 Hz), 122.07 (q, *J* = 2.9 Hz), 121.35, 120.81 (Figure S23); HR-MS: C_16_H_10_ONBrF_3_ [M − H]^−^ calculated 367.9903 *m/z*, found 367.9893 *m/z* (Figure S24).

*(2E)-N-[4-Fluoro-2-(trifluoromethyl)phenyl]-3-phenylprop-2-enamide* (**14**), Yield 76%; Mp 145–147 °C; IR (cm^−1^): 3257, 3086, 1653, 1622, 1521, 1492, 1429, 1332, 1316, 1271, 146, 1174, 1127, 1119, 1049, 974, 914, 883, 865, 845, 823, 761, 745, 727, 709, 691, 664, 652, 569, 539, 499, 481; ^1^H-NMR (DMSO-*d_6_*), δ: 9.79 (s, 1H), 7.67 (dd, *J* = 8.9 Hz, *J* = 2.7 Hz, 1H), 7.65–7.63 (m, 2H), 7.61–7.58 (m, 2H), 7.59 (d, *J* = 15.8 Hz, 1H), 7.46–7.40 (m, 3 H), 6.97 (d, *J* = 15.8 Hz, 1H); ^13^C-NMR (DMSO-*d_6_*), δ: 164.79, 159.51 (d, *J* = 245.7 Hz), 140.86, 134.55, 132.54 (d, *J* = 8.7 Hz), 131.76 (d, *J* = 2.9 Hz), 129.92, 129.02, 127.81, 126.40 (qd, *J* = 31.8 Hz, *J* = 10.1 Hz), 122.67 (qd, *J* = 274.6 Hz, *J* = 2.9 Hz), 121.18, 120.00 (d, *J* = 21.7 Hz), 113.72 (dq, *J* = 26.0 Hz, *J* = 5.8 Hz) (Figure S25); HR-MS: C_16_H_10_ONF_4_ [M − H]^−^ calculated 308.0704 *m/z*, found 308.0696 *m/z* (Figure S26).

*(2E)-N-[4-Chloro-2-(trifluoromethyl)phenyl]-3-phenylprop-2-enamide* (**15**), Yield 71%; Mp 165–167 °C; IR (cm^−1^): 3268, 3083, 3030, 1655, 1624, 1584, 1522, 1483, 1449, 1410, 1337, 1306, 1277, 1268, 1243, 1171, 1122, 1109, 1052, 967, 890, 872, 857, 833, 810, 761, 711, 696, 684, 562, 539, 509; ^1^H-NMR (DMSO-*d_6_*), δ: 9.80 (s, 1H), 7.84 (d, *J* = 2.1 Hz, 1H), 7.79 (dd, *J* = 8.6 Hz, *J* = 2.4 Hz, 1H), 7.69 (d, *J* = 8.9 Hz, 1H), 7.65–7.64 (m, 2H), 7.60(d, *J* = 15.8 Hz, 1H), 7.47–7.41 (m, 3H), 7.00 (d, *J* = 15.8 Hz, 1H); ^13^C-NMR (DMSO-*d_6_*). δ: 164.65, 141.13, 134.52, 134.38, 132.96, 131.51, 130.67, 129.98, 129.03, 127.86, 126.26 (q, *J* = 5.8 Hz), 125.72 (q, *J* = 30.3 Hz), 122.7 (q, *J* = 273.1 Hz), 121.12 (Figure S27); HR-MS: C_16_H_10_ONClF_3_ [M − H]^−^ calculated 324.0409 *m/z*, found 324.0399 *m/z* (Figure S28).

*(2E)-N-[4-Bromo-2-(trifluoromethyl)phenyl]-3-phenylprop-2-enamide* (**16**), Yield 64%; Mp 176–178 °C; IR (cm^−1^): 3289, 3083, 3030, 1657, 1624, 1525, 1481, 1404, 1338, 1305, 1278, 1265, 1247, 1172, 1160, 1124, 1052, 967, 889, 866, 831, 760, 747, 710, 682, 631, 560, 529, 501, 463; ^1^H-NMR (DMSO-*d_6_*), δ: 9.78 (s, 1H), 7.94 (m, 1H), 7.92 (dd, *J* = 8.2 Hz, *J* = 2.1 Hz, 1H), 7.65–7.62 (m, 3H), 7.60 (d, *J* = 15.8 Hz, 1H), 7.46–7.41 (m, 3H), 7.00 (d, *J* = 15.8 Hz, 1H); ^13^C-NMR (DMSO-*d_6_*), δ: 164.59, 141.16, 135.94, 134.81 (q, *J* = 2.9 Hz), 134.52, 131.64, 129.99, 129.03, 129.02 (q, *J* = 5.8 Hz), 127.86, 125.87 (q, *J* = 30.3 Hz), 122.60 (q, *J* = 273.1 Hz), 121.12, 118.62 (Figure S29); HR-MS: C_16_H_10_ONBrF_3_ [M − H]^−^ calculated 367.9903 *m/z*, found 367.9894 *m/z* (Figure S30).

*(2E)-N-[4-Nitro-2-(trifluoromethyl)phenyl]-3-phenylprop-2-enamide* (**17**), Yield 61%; Mp 164–166 °C; IR (cm^−1^): 3314, 3086, 1662, 1620, 1594, 1544, 1505, 1449, 1424, 1338, 1319, 1277, 1172, 1112, 1052, 1000, 973, 921, 901, 854, 840, 790, 760, 744, 709, 686, 604, 592, 560; ^1^H-NMR (DMSO-*d_6_*), δ: 9.95 (s, 1H), 8.54 (dd, *J* = 8.9 Hz, *J* = 2.7 Hz, 1H), 8.47 (d, *J* = 2.7 Hz, 1H), 8.17 (d, *J* = 8.9 Hz, 1H), 7.68 (d, *J* = 15.8 Hz, 1H), 7.68–7.67 (m, 2H), 7.48–7.43 (m, 3H), 7.17 (d, *J* = 15.8 Hz, 1H); ^13^C-NMR (DMSO-*d_6_*), δ: 164.64, 143.84, 142.29, 141.30, 134.41, 130.25, 129.06, 128.84, 128.04, 127.96, 122.50 (q, *J* = 30.3 Hz), 122.48 (q, *J* = 273.10 Hz), 122.27 (q, *J* = 4.3 Hz), 120.91 (Figure S31); HR-MS: C_16_H_10_O_3_N_2_F_3_ [M − H]^−^ calculated 335.0649 *m/z*, found 335.0637 *m/z* (Figure S32).

*(2E)-N-[4-Nitro-3-(trifluoromethyl)phenyl]-3-phenylprop-2-enamide* (**18**), Yield 60%; Mp 181–183 °C; IR (cm^−1^): 3314, 3086, 1662, 1620, 1594, 1544, 1505, 1424, 1338, 1319, 1277, 1247, 1172, 1112, 1052, 983, 921, 901, 854, 840, 790, 760, 750, 709, 686, 645, 604, 593, 560, 505; ^1^H-NMR (DMSO-*d_6_*), δ: 11.03 (s, 1H), 8.38 (d, *J* = 2.1 Hz, 1H), 8.23 (d, *J* = 8.9 Hz, 1H), 8.13 (dd, *J* = 8.9 Hz, *J* = 2.1 Hz, 1H), 7.70 (d, *J* = 15.8 Hz, 1H),7.67–7.66 (m, 2H), 7.48–7.43 (m, 3H), 6.81 (d, *J* = 15.8 Hz, 1H); ^13^C-NMR (DMSO-*d_6_*), δ: 164.54, 143.95, 142.27, 141.30, 134.23, 130.35, 129.10, 128.04, 127.84, 123.02 (q, *J* = 33.2 Hz), 122.07 (q, *J* = 273.1 Hz), 120.94, 117.32 (q, *J* = 5.8 Hz) (Figure S33); HR-MS: C_16_H_10_O_3_N_2_F_3_ [M − H]^−^ calculated 335.0649 *m/z*, found 335.0635 *m/z* (Figure S34).

*(2E)-N-[2,6-Dibromo-4-(trifluoromethyl)phenyl]-3-phenylprop-2-enamide* (**19**), Yield 78%; Mp 227–229 °C; IR (cm^−1^): 3086, 2970, 2855, 1655, 1613, 1520, 1450, 1395, 1340, 1303, 1197, 1167, 1132, 1096, 1072, 1010, 984, 881, 862, 768, 743, 715, 692, 666, 566, 497; ^1^H-NMR (DMSO-*d_6_*), δ: 10.36 (s, 1H), 8.19 (s, 2H), 7.67–7.66 (m, 2H), 7.64 (d, *J* = 15.8 Hz, 1H), 7.47–7.42 (3H), 6.90(d, *J* = 15.8 Hz, 1H); ^13^C-NMR (DMSO-*d_6_*), δ: 163.42, 141.14, 140.13, 134.38, 130.08, 129.99 (q, *J* = 33.2 Hz), 129.16 (q, *J* = 4.3 Hz), 129.07, 127.88, 124.96, 122.29 (q, *J* = 273.1 Hz), 120.45 (Figure S35); HR-MS: C_16_H_9_ONBr_2_F_3_ [M − H]^−^ calculated 445.9008 *m/z*, found 445.9002 *m/z* (Figure S36).

*(2E)-N-(2,6-Dibromo-3-chloro-4-fluorophenyl)-3-phenylprop-2-enamide* (**20**), Yield: 74%; Mp 234–235 °C; IR (cm^−1^):3197, 3001, 1656, 1664, 1611, 1571, 1523, 1442, 1357, 1339, 1296, 1285, 1206, 1179, 1113, 988, 979, 854, 784, 763, 753, 723, 696, 684, 640, 581, 562, 530, 486, 442; ^1^H-NMR (DMSO-*d_6_*), δ: 10.26 (s, 1H), 8.07 (d, *J* = 8.9 Hz, 1H), 7.67–7.65 (m, 2H), 7.62 (d, *J* = 15.8 Hz, 1H), 7.47–7.42 (m, 3H), 6.87 (d, *J* = 15.8 Hz, 1H); ^13^C-NMR (DMSO-*d_6_*), δ: 163.64, 156.15 (d, *J* = 252.9 Hz), 141.25, 134.40, 134.28 (d, *J* = 2.9 Hz), 130.04, 129.07, 127.85, 126.30, 122.82 (d, *J* = 10.1 Hz), 121.30 (d, *J* = 20.2 Hz), 120.52, 119.79 (d, *J* = 24.6 Hz) (Figure S37); HR-MS: C_15_H_8_ONBr_2_FCl [M − H]^−^ calculated 429.8651 *m/z*, found 429.8648 *m/z* (Figure S38).

### 3.2. Biological Testing

#### 3.2.1. In Vitro Antibacterial Evaluation

The synthesized compounds were evaluated for their in vitro antibacterial activity against representatives of multidrug-resistant bacteria, clinical isolates of methicillin-resistant *Staphylococcus aureus* (MRSA) 63718, and SA 630 that were obtained from the National Institute of Public Health (Prague, Czech Republic). *S. aureus* ATCC 29213 was used as the reference and quality control strain. Ampicillin (Sigma, St. Louis, MO, USA) was employed as the standard. All compounds and controls were prepared in triplicate. The screening was performed as described previously [26]. The results are summarized in Table 1. Antibacterial activity was assessed by determining MIC and MBC values. The broth microdilution method was applied. MIC values were determined in a microtiter plate. Aliquots from this plate were subcultivated on the Petri dish with Mueller-Hinton broth for the determination of MBC [43].

#### 3.2.2. In Vitro Antimycobacterial Evaluation

The assessment of the in vitro antimycobacterial activity of the compounds was performed against *Mycobacterium tuberculosis* H37Ra/ATCC 25177 by means of the methodology described recently (e.g., [26]). Isoniazid (Sigma) was used as the standard. All compounds and controls were prepared in triplicate. The results are summarized in Table 1.

#### 3.2.3. MTT Assay

A compound was diluted in Mueller-Hinton broth to achieve the desired final concentrations of 0.5 and 1 µg/mL, respectively. *S. aureus* ATCC 29213 bacterial suspension in sterile distilled water at 0.5 McFarland was diluted 1:3. Inocula were added to each well by a multi-inoculator. Diluted bacteria in broth free from inhibiting compounds were used as the growth control. The procedure was performed three times. Plates were incubated at 37 °C for 24 h. After the incubation period, 10% well volume of MTT (3-(4,5-dimethylthiazol-2-yl)-2,5-diphenyltetrazolium bromide) reagent (Sigma) was mixed into each well and incubated at 37 °C for 1 h. Then 100 µL of 17% sodium dodecyl sulphate in 40% dimethylformamide was added to each well. The plates were read at 570 nm. The absorbance readings from the cells grown in the presence of the tested compounds were compared with uninhibited cell growth to determine relative percent inhibition. The percent inhibition was determined through the MTT assay. The percent viability is calculated through the comparison of a measured value and that of the uninhibited control: % viability = OD_570E_/OD_570P_ × 100, where OD_570E_ is the reading from the compound-exposed cells, while OD_570P_ is the reading from the uninhibited cells (positive control). Cytotoxic potential is determined by a percent viability of <70% [43,59].

#### 3.2.4. In Vitro Cell Viability Assay

The cytotoxic effect of the tested compounds was specified on THP1-Blue™ NF-κB cell line (Invivogen; San Diego, CA, USA), as described previously [28]. Briefly, cells resuspended in serum-free RPMI 1640 medium (Merck, Darmstadt, Germany) supplemented with antibiotics (100 U/mL penicillin and 100 mg/mL streptomycin (Merck)) and 10% FBS (Merck) were seeded into 96-well plates (100 µL/well, i.e., 50,000 cells per each well). After 2 h, tested compounds dissolved in DMSO (1.25–20 µM) were added to the cells. The final concentration of DMSO was 0.1% (*v/v*) in each well. The viability analysis was performed after 24 h incubation with the tested substances using the WST-1 Cell Proliferation Reagent kit (Roche Diagnostics, Basel, Switzerland) according to the manufacturer’s manual. The amount of formazan formed, which corresponded to the number of metabolically active cells in the culture, was calculated as a percentage of the control cells, which were treated only with serum-free RPMI 1640 medium and were assigned as 100%. The IC_50_ values were calculated by four-parameter logistic (4PL) analysis from obtained viability curves by GraphPad Prism 8.0.1 (San Diego, CA, USA) software. The results are summarized in Table 1 as mean ± SEM (n = 6).

#### 3.2.5. Determination of NF-κB Activity

To evaluate the anti-inflammatory potential of novel compounds, their ability to attenuate the lipopolysaccharide (LPS)-activated pro-inflammatory transcription nuclear factor (NF)-κB was measured as was described previously [28]. Briefly, THP1-Blue™ NF-κB cells were pretreated by tested compounds dissolved in DMSO in the non-toxic concentration 1 µM for 1 h. After LPS (1 µg/mL) stimulation, the NF-κB activity was evaluated by Quanti-Blue medium (Invivogen) according to the manufacturer’s instructions. The results from 3 independent experiments performed in triplicate (n = 9) were analyzed by GraphPad Prism 8.0.1 software. The outlaying values were excluded by ROUT algorithms (Q = 5%).

### 3.3. Lipophilicity Determination by RP-HPLC (Capacity Factor k/Calculated log k)

The HPLC separation system Merck Hitachi LaChrom Elite^®^ equipped with a Merck Hitachi LaChrom Elite^®^ L-2455 Diode-Array Detector (Hitachi High Technologies America, San Jose, CA, USA) was used. A chromatographic column Symmetry^®^ C18 5 μm, 4.6 × 250 mm, Part No. W21751W016 (Waters Corp., Milford, MA, USA) was used. The HPLC separation process was monitored by the EZ CHROM Elite^®^ software (Hitachi High Technologies America). The total flow of the column was 1.0 mL/min, injection 10 μL, column temperature 40 °C, and sample temperature 10 °C. The detection wavelength 214 nm was chosen. A KI methanolic solution was used for the dead time (*t_d_*) determination. Retention times (*t_r_*) were measured in minutes. Isocratic elution by a mixture of Metanol Chromasolv^TM^ (Honeywell, St. Louis, MO, USA) (72%) and H_2_O-HPLC Mili-Q grade (Labconco, Kansas City, MO, USA) (28%) as a mobile phase was used for the determination of capacity factor *k*, while isocratic elution by a mixture of Metanol Chromasolv^TM^ (Honeywell) (72%) and NaOAc buffer, pH 6.5 and 7.4 (28%) as a mobile phase were used for the determination of distribution coefficients *D*_6.5_ and *D*_7.4_. The capacity factors and distribution coefficients were calculated according to the formula *k*(*D*) = (*t_r_* − *t_d_*)/*t_d_*, where *t_R_* is the retention time of the solute and *t_d_* is the dead time obtained using an unretained analyte. Each experiment was repeated three times. The calculated log *k*, log *D*_6.5_, and log *D*_7.4_ values of individual compounds are shown in Table 1.

### 3.4. Lipophilicity Determination by RP-TLC

R_m_ values were determined from the RP-18 TLC measurements. The solutions of compounds in Metanol Chromasolv^TM^ (Honeywell) were spotted on a RP-TLC plate (Silica gel Nano-SIL C18-100 UV 254, 10 × 10 cm, Macherey-Nagel, Duren, Germany), 1.5 cm from the edge. The volatiles were carefully evaporated, and the plate was developed by MeOH (100%) or MeOH: H_2_O-HPLC (72:28 *v*/*v*) or MeOH: NaOAc buffer, pH 7.4 (72:28 *v*/*v*). After drying, the spots were visualized under UV (λ = 365 nm). R_m_ data were obtained from equation: R_m_ = log(1/R_f_-1). Each experiment was repeated three times. The R_m_ values of individual compounds are shown in Table 1.

### 3.5. Model Building and Experimental vs. Theoretical Lipophilicity Prediction

CACTVS/csed and CORINA editors were engaged to produce each structural model and its initial spatial geometry. OpenBabel (inter)change file format converter was employed for data conversion. Sybyl-X 2.0/Certara software package running on a HP Z200 workstation with a Debian 10.0 operating system was used to conduct the molecular modeling simulations. The initial compound geometry optimization with MAXMIN2 module was performed using the standard Tripos force field (POWELL conjugate gradient algorithm) with a 0.01 kcal/mol energy gradient convergence criterion. The specification of the electrostatic potential values based on the partial atomic charges was carried out with the Gasteiger–Hückel method implemented in Sybyl-X.

An ensemble of freely/commercially available in silico predictors can be engaged to specify theoretically the numerical value of partition coefficients (clogP) as follows:

AlogPS—algorithm implemented by Tetko et al. [37] based on atom-type electrotopological-state (E-state) indices and neural networks (NN);

milogP—procedure proposed by Molinspiration for practical logP calculations of almost all organic molecules as a sum of fragment-based contributions and correction factors;

ClogP—fragment-based approach for estimation of lipophilicity based on structure-dependent correction values retrieved from Hansch and Leo’s database that is implemented in Sybyl/Centara software;

HyperChem logP—an atom-additive method that estimates lipophilicity using the individual atomic contribution based on procedure proposed by Ghose, Prichett and Crippen;

MarvinSketch logP—the overall lipophilicity of a molecule is composed of the contributing values of its atom types that were redefined to accommodate electron delocalization and contributions of ionic forms;

ChemSketch logP—a comprehensive fragment-based algorithm with high quality models built using empirical data. Well-characterized logP contributions have been compiled for atoms; structural fragments and intramolecular interactions provided for more than 12 × 10^3^ experimental logP values;

Dragon AlogP—statistical estimators of the Ghose-Crippen-Viswanadhan model were specified on the basis of known experimental logP for the training set of 8364 compounds. The overall approach of the lipophilic atomic-based constant is estimated with the contribution of 115 atom types;

Dragon MlogP—the calculated partition coefficient includes VdW volume and Moriguchi polar parameters as correction factors. A regression MlogP model is based on 13 structural parameters that were rated on the training group of 1,230 organic molecules;

Kowwin—estimates the log octanol-water partition coefficient of chemicals using the atom/fragment contribution protocol;

XlogP3—an atom-additive procedure with well-defined correction factors that engages an optimized atom typing approach calibrated on a big training set;

OSIRIS clogP—in house approach based on the cumulative sum of atom contributions calculated for more than 5000 compounds with experimentally measured logP values as a training set. Predicting engine distinguishes 369 atom types;

ChemDraw clogP—the combination of Classic and GALAS algorithms for the prediction of partition coefficient based on a training set of >11 × 10^3^ compounds provides coverage for a broad chemical space;

Percepta clogP—based on >12 × 10^3^ of empirical logP values with the algorithm that uses the principal of isolating carbons.

In order to detect the redundant descriptors in QSAR/QSPR studies, several methods of variable selection/elimination have been proposed, including the uninformative variable elimination (UVE–PLS), as well as its modifications, namely iterative variable elimination (IVE–PLS) [60]. Overall, the entire algorithm comprises of the following stages:

Stage 1. Standard PLS analysis with LOO–CV to evaluate the performance of the PLS model

Stage 2. Elimination of the matrix column with the lowest abs(mean(b)/std(b)) value

Stage 3. Standard PLS analysis of the new matrix without the column eliminated in Stage 2.

Stage 4. Iterative repetition of the Stages 1–3 to maximize the LOO $q_{cv}^{2}$.

### 3.6. Similarity Assessment Using PCA and Tanimoto Coefficient

The proper mapping of the molecular diversity in the theoretically infinite chemical space (CS ≈ 10^60^–10^200^) into the corresponding property space basically requires the multi-dimensional description of a molecule by a set of structural (S) and physicochemical (P) properties organized in a vector [61]. The molecular distribution of the experimental-based (FCS) and virtual-derived (VCS) compounds/models might be graphically displayed using the human friendly 2D/3D plots, e.g., in the procedure called Principal Component Analysis (PCA) [62]. PCA is a linear projection method designed to model multivariate data with a relatively small number of so-called principal components (scores and loadings) constructed in order to maximize the variance description of input data [63]. The PCA model with *f* principal components for a data matrix *X* can be presented as follows:

*X* = *TP^T^* + *E* (1)

where *X* is a data matrix with *m* objects and *n* variables, *T* is the score matrix with dimensions (*m* × *f*), *P^T^* is a transposed matrix of loadings with dimensions (*f* × *n*) and *E* is a matrix of the residual variance (*m* × *n*) that is not explained by the first *f* principal components. Basically, the first few principal components often capture interesting information about the data structure and uncover groups of objects.

The structural relatedness between molecule library is usually estimated using a function mapping (dis)similarities between the pairs of bit-string descriptors given by the Tanimoto equation as follows:

(2) $$T(x,y)=\frac{n_{xy}}{\left(n_{x}+n_{y}-n_{xy}\right)}$$

where $n_{xy}\;$ is the number of bits set into 1 shared in the fingerprint of molecules *x* and *y*, $n_{x}$ is the number of bits set into 1 in molecule *x*, $n_{y}$ is the number of bits set into 1 in molecule *y*. Top-ranked objects are supposed to have similar properties, although the validity of this assumption is fairly questionable, because 70% of compounds with T(x,y) > 0.85 to an active analog have comparatively low probability of being active in the same way [64].

## 4. Conclusions

A set of 20 cinnamic acid anilides (1 published recently and 19 newly synthesized) was investigated in relation to their biological activities and characterized by a series of experimental lipophilicity values generated using RP-HPLC and RP-TLC methods. All compounds were tested against the reference strain *Staphylococcus aureus*, two MRSA clinical isolates, and *Mycobacterium tuberculosis*. (2*E*)-*N*-[3-Fluoro-4-(trifluoromethyl)phenyl]-3-phenylprop-2-enamide (**10**) showed comparable or even better activity than the ampicillin. It was found that the activity of compound **10** is bactericidal. The screening of the cell viability performed using THP1-Blue™ NF-κB cells demonstrated that only (2*E*)-*N*-(4-bromo-3-chlorophenyl)-3-phenylprop-2-enamide (**11**) showed significant cytotoxic effect. Moreover, all compounds were tested for their anti-inflammatory potential; (2*E*)-*N*-(2,6-dibromo-3-chloro-4-fluorophenyl)-3-phenylprop-2-enamide (**20**) attenuated the lipopolysaccharide-induced NF-κB activation and was more potent than the parental cinnamic acid. Unfortunately, the design of compound that combines both high antimicrobial activity and anti-inflammatory effect is a great challenge; compounds with preferential anilide ring substitution at the *meta* positions and optionally in combination with *para* show the highest antimicrobial activity, while the *ortho* position optionally in combination with *meta* are anti-inflammatory preferential. The similarity-related property space evaluation for the congeneric series of structural analogues was carried out using the principal component analysis (PCA). Moreover, the in silico approximation of the lipophilic values for the ensemble of anilides **1**–**20** was performed employing a set of free/commercial clogP estimators, subsequently corrected using the corresponding pK_a_ calculated at physiological pH and subsequently cross-compared with the experimental parameter. The mean and median value of the selected estimators that averaged over the chosen logD_7.4_ values were subsequently correlated with the experimental parameter with correlation coefficient of 0.65, because not only the best inter-correlated clogD_7.4_ values were specified in the consensus clogP approach. Finally, the similarity-driven investigation using Tanimoto metrics was conducted revealing the structural dissimilarities of nitro-substituted isomers (compounds **17** (R = 2-CF_3_-4-NO_2_) and **18** (R = 3-CF_3_-4-NO_2_)) as compared with the remaining ones, which confirms our previous PCA findings. Interestingly, mono-bromo/chloro-substituted isomers (compounds **11** (R = 3-Cl-4-Br) and **12** (R = 2-Br-4-Cl)) indicate the structural similarity to di-/tri-bromo/chloro-substituted positional isomers (molecules **4**–**9**). Unfortunately, the similarity investigation did not provide valuable hints that could explain the noticeable variations in the toxic effect exerted by molecule **11** (IC_50_ = 6.5 µM on THP1-Blue™ NF-κB cells) and the remaining ones. In general, the distribution coefficient provides a more realistic estimation of lipophilicity in the relevant pH environments; therefore, logD_pH_ ought to be preferentially engaged in the QSPR study, especially for compounds that are likely to ionize in physiological media. Based on this study, the antiproliferative/antineoplastic activity of compound **11** will be investigated, while the rest of the tested compounds will be studied in relation to their antimicrobial and anti-inflammatory activities.

## Figures and Tables

**Table 1:** Structure of ring-substituted (2*E*)-*N*-aryl-3-phenylprop-2-enamides **1**–**20**, in vitro anti-*Staphylococcus* activities’ minimum inhibitory concentration (MIC; μM) in comparison with standard ampicillin (AMP), in vitro antitubercular activity MIC (μM) in comparison with standard isoniazid (INH), and their influence on viability of THP1-Blue™ NF-κB cell line (IC_50_ (µM) ± SEM, n = 6).

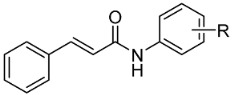						
Comp.	R	MIC (μM)				Tox IC_50_ (µM) ± SEM
		SA	MRSA 63718	MRSA SA 630	Mtb	
1 *	H	>1146	>1146	>1146	286	>20
**2**	2,4,6-F	>924	>924	>924	>462	>20
**3**	3,4,5-F	462	>924	>924	>462	>20
**4**	2,4-Cl	>880	>880	>880	>440	>20
**5**	2,4,5-Cl	>790	>790	>790	>395	>20
**6**	3,4,5-Cl	>790	>790	>790	>395	>20
**7**	2,4-Br	>677	>677	>677	>339	>20
**8**	2-F-5-Cl	931	>931	>931	>465	>20
**9**	3-F-4-Br	>805	>805	805	>403	>20
**10**	3-F-4-CF_3_	25.9	25.9	12.9	>414	>20
**11**	3-Cl-4-Br	>766	192	192	>383	6.5 ± 1.0
**12**	2-Br-4-Cl	>766	>766	>766	>383	>20
**13**	2-Br-5-CF_3_	>696	>696	>696	>348	>20
**14**	2-CF_3_-4-F	>828	>828	>828	>414	>20
**15**	2-CF_3_-4-Cl	>788	>788	>788	>394	>20
**16**	2-CF_3_-4-Br	>696	>696	>696	>348	>20
**17**	2-CF_3_-4-NO_2_	>762	>762	>762	>381	>20
**18**	3-CF_3_-4-NO_2_	>762	>762	>762	>381	>20
**19**	2,6-Br-4-CF_3_	>574	>574	>574	>287	>20
**20**	2,6-Br-3-Cl-4-F	>595	>595	>595	>298	>20
**AMP**	–	5.72	45.8	45.8	–	–
**INH**	–	–	–	–	36.6	–

* compound was described in reference [26]; SA *= Staphylococcus aureus* ATCC 29213; MRSA *=* clinical isolates of methicillin-resistant *S. aureus* 63,718 and SA 630 (National Institute of Public Health, Prague, Czech Republic); Mtb = *Mycobacterium tuberculosis* H37Ra.

**Table 2:** Experimentally found lipophilicity determined using HPLC and TLC approaches for *N*-arylcinnamanilides **1**–**20**.

Comp.	R	RP-HPLC			RP-TLC		
		Logk ^a^	logD ^b^	logD ^c^	R_M_^d^	R_M_^e^	R_M_^f^
**1**	H	0.0270	0.1251	0.1090	−0.5087	0.2848	0.4038
**2**	2,4,6-F	−0.0831	0.0260	0.0107	−0.6536	0.1218	0.1951
**3**	3,4,5-F	0.4893	0.5495	0.5274	−0.4206	0.6074	0.5495
**4**	2,4-Cl	0.5278	0.5885	0.5685	−0.3933	0.6546	0.5495
**5**	2,4,5-Cl	0.8373	0.8879	0.8647	−0.2497	0.9695	0.9832
**6**	3,4,5-Cl	0.9671	1.0104	0.9897	−0.1870	1.0852	1.1306
**7**	2,4-Br	0.6152	0.6704	0.6518	−0.3098	0.7331	0.7295
**8**	2-F-5-Cl	0.3692	0.4334	0.4154	−0.4711	0.5411	0.5495
**9**	3-F-4-Br	0.5025	0.5597	0.5408	−0.4081	0.6797	0.6895
**10**	3-F-4-CF_3_	0.5789	0.6327	0.6095	−0.5032	0.7923	0.5960
**11**	3-Cl-4-Br	0.6611	0.7126	0.6912	−0.3376	0.8388	0.8181
**12**	2-Br-4-Cl	0.5476	0.6064	0.5860	−0.4072	0.6553	0.6484
**13**	2-Br-5-CF_3_	0.5561	0.6157	0.5936	−0.4325	0.7194	0.5619
**14**	2-CF_3_-4-F	0.1607	0.2426	0.2220	−0.6797	0.3011	0.1330
**15**	2-CF_3_-4-Cl	0.4113	0.4741	0.454	−0.6102	0.5305	0.3219
**16**	2-CF_3_-4-Br	0.4683	0.5316	0.5105	−0.5624	0.5730	0.4273
**17**	2-CF_3_-4-NO_2_	0.3794	0.4431	0.3989	−0.5633	0.4998	0.4311
**18**	3-CF_3_-4-NO_2_	0.5004	0.5571	0.5098	−0.4804	0.6927	0.6160
**19**	2,6-Br-4-CF_3_	0.4321	0.4941	0.4731	−0.5206	0.5301	0.3733
**20**	2,6-Br-3-Cl-4-F	0.3139	0.3922	0.3699	−0.5541	0.4320	0.4138

^a^ 72:28 MeOH:H_2_O; ^b^ 72:28 MeOH:NaOAc buffer (pH 7.4), ^c^ 72:28 MeOH: NaOAc buffer (pH 6.5), ^d^ 100% MeOH, ^e^ 72:28 MeOH:H_2_O, ^f^ 72:28 MeOH: NaOAc buffer (pH 7.4).

## Supplementary Materials

The following are available online, Table S1: Theoretically estimated partition coefficient calculated by set of alternative methods for anilides **1**–**20**. Table S2: Theoretically estimated pK_a_ calculated by ACD/Percepta/pK_a_ for anilides **1**–**20**. Figure S1: ^13^C-NMR (DMSO-*d_6_*) spectrum of (2*E*)-3-phenyl-*N*-(2,4,6-trifluorophenyl)prop-2-enamide (**2**). Figure S2: HR-MS record of (2*E*)-3-phenyl- *N*-(2,4,6-trifluorophenyl)prop-2-enamide (**2**). Figure S3: ^13^C-NMR (DMSO-*d_6_*) spectrum of (2*E*)-3-phenyl- *N*-(3,4,5-trifluorophenyl)prop-2-enamide (**3**). Figure S4: HR-MS record of (2*E*)-3-phenyl-*N*-(3,4,5-trifluorophenyl)- prop-2-enamide (**3**). Figure S5: ^13^C-NMR (DMSO-*d_6_*) spectrum of (2*E*)-*N*-(2,4-dichlorophenyl)-3-phenylprop- 2-enamide (**4**). Figure S6: HR-MS record of (2*E*)-*N*-(2,4-dichlorophenyl)-3-phenylprop-2-enamide (**4**). Figure S7: ^13^C-NMR (DMSO-*d_6_*) spectrum of (2*E*)-3-phenyl-*N*-(2,4,5-trichlorophenyl)prop-2-enamide (**5**). Figure S8: HR-MS record of (2*E*)-3-phenyl-*N*-(2,4,5-trichlorophenyl)prop-2-enamide (**5**). Figure S9: ^13^C-NMR (DMSO-*d_6_*) spectrum of (2*E*)-3-phenyl-*N*-(3,4,5-trichlorophenyl)prop-2-enamide (**6**). Figure S10: HR-MS record of (2*E*)-3-phenyl- *N*-(3,4,5-trichlorophenyl)prop-2-enamide (**6**). Figure S11: ^13^C-NMR (DMSO-*d_6_*) spectrum of (2*E*)-*N*-(2,4-dibromo- phenyl)-3-phenylprop-2-enamide (**7**). Figure S12: HR-MS record of (2*E*)-*N*-(2,4-dibromophenyl)-3-phenylprop- 2-enamide (**7**). Figure S13: ^13^C-NMR (DMSO-*d_6_*) spectrum of (2*E*)-*N*-(5-chloro-2-fluorophenyl)-3-phenylprop- 2-enamide (**8**). Figure S14: HR-MS record of (2*E*)-*N*-(5-chloro-2-fluorophenyl)-3-phenylprop-2-enamide (**8**). Figure S15: ^13^C-NMR (DMSO-*d_6_*) spectrum of (2*E*)-*N*-(4-bromo-3-fluorophenyl)-3-phenylprop-2-enamide (**9**). Figure S16: HR-MS record of (2*E*)-*N*-(4-bromo-3-fluorophenyl)-3-phenylprop-2-enamide (**9**). Figure S17: ^13^C-NMR (DMSO-*d_6_*) spectrum of (2*E*)-*N*-[3-fluoro-4-(trifluoromethyl)phenyl]-3-phenylprop-2-enamide (**10**). Figure S18: HR-MS record of (2*E*)-*N*-[3-fluoro-4-(trifluoromethyl)phenyl]-3-phenylprop-2-enamide (**10**). Figure S19: ^13^C-NMR (DMSO-*d_6_*) spectrum of (2*E*)-*N*-(4-bromo-3-chlorophenyl)-3-phenylprop-2-enamide (**11**). Figure S20: HR-MS record of (2*E*)-*N*-(4-bromo-3-chlorophenyl)-3-phenylprop-2-enamide (**11**). Figure S21: ^13^C-NMR (DMSO-*d_6_*) spectrum of (2*E*)-*N*-(2-bromo-4-chlorophenyl)-3-phenylprop-2-enamide (**12**). Figure S22: HR-MS record of (2*E*)-*N*-(2-bromo-4-chlorophenyl)-3-phenylprop-2-enamide (**12**). Figure S23: ^13^C-NMR (DMSO-*d_6_*) spectrum of (2*E*)-*N*-[2-bromo-5-(trifluoromethyl)phenyl]-3-phenylprop-2-enamide (**13**). Figure S24: HR-MS record of (2*E*)-*N*-[2-bromo-5-(trifluoromethyl)phenyl]-3-phenylprop-2-enamide (**13**). Figure S25: ^13^C-NMR (DMSO-*d_6_*) spectrum of (2*E*)-*N*-[4-fluoro-2-(trifluoromethyl)phenyl]-3-phenylprop-2-enamide (**14**). Figure S26: HR-MS record of (2*E*)-*N*-[4-fluoro-2-(trifluoromethyl)phenyl]-3-phenylprop-2-enamide (**14**). Figure S27: ^13^C-NMR (DMSO-*d_6_*) spectrum of (2*E*)-*N*-[4-chloro-2-(trifluoromethyl)phenyl]-3-phenylprop-2-enamide (**15**). Figure S28: HR-MS record of (2*E*)-*N*-[4-chloro-2-(trifluoromethyl)phenyl]-3-phenylprop-2-enamide (**15**). Figure S29: ^13^C-NMR (DMSO-*d_6_*) spectrum of (2*E*)-*N*-[4-bromo-2-(trifluoromethyl)phenyl]-3-phenylprop-2-enamide (**16**). Figure S30: HR-MS record of (2*E*)-*N*-[4-bromo-2-(trifluoromethyl)phenyl]-3-phenylprop-2-enamide (**16**). Figure S31: ^13^C-NMR (DMSO-*d_6_*) spectrum of (2*E*)-*N*-[4-nitro-2-(trifluoromethyl)phenyl]-3-phenylprop-2-enamide (**17**). Figure S32: HR-MS record of (2*E*)-*N*-[4-nitro-2-(trifluoromethyl)phenyl]-3-phenylprop-2-enamide (**17**). Figure S33: ^13^C-NMR (DMSO-*d_6_*) spectrum of (2*E*)-*N*-[4-nitro-3-(trifluoromethyl)phenyl]-3-phenylprop-2-enamide (**18**). Figure S34: HR-MS record of (2*E*)-*N*-[4-nitro-3-(trifluoromethyl)phenyl]-3-phenylprop-2-enamide (**18**). Figure S35: ^13^C-NMR (DMSO-*d_6_*) spectrum of (2*E*)-*N*-[2,6-dibromo-4-(trifluoromethyl)phenyl]-3-phenylprop-2-enamide (**19**). Figure S36: HR-MS record of (2*E*)-*N*-[2,6-dibromo-4-(trifluoromethyl)phenyl]-3-phenylprop-2-enamide (**19**). Figure S37: ^13^C-NMR (DMSO-*d_6_*) spectrum of (2*E*)-*N*-(2,6-dibromo-3-chloro-4-fluorophenyl)-3-phenylprop-2-enamide (**20**). Figure S38: HR-MS record of (2*E*)-*N*-(2,6-dibromo-3-chloro-4-fluorophenyl)-3-phenylprop-2-enamide (**20**).
