# Supplementary material for: Biological Activities and ADMET-Related Properties of Novel Set of Cinnamanilides
Source: Molecules. 2020 Sep 9;25(18):4121. doi: 10.3390/molecules25184121 (PMC7570544; doi:10.3390/molecules25184121)
Supplement: Supplementary file 1 [file molecules-25-04121-s001.pdf]

Supplementary Materials

Biological Activities and ADMET-Related Properties of Novel Set of Cinnamanilides †

Jiri Kos [1], Andrzej Bak [2,*], Violetta Kozik [2], Timotej Jankech [3], Tomas Strharsky [1], Aleksandra Swietlicka [2], Hana Michnova [1], Jan Hosek [1], Adam Smolinski [4], Michal Oravec [5], Ferdinand Devinsky [6], Milan Hutta [3] and Josef Jampilek [3,*]

[1] Regional Centre of Advanced Technologies and Materials, Faculty of Science, Palacky University, Slechtitelu 27, 78371 Olomouc, Czech Republic; jiri.kos@upol.cz (J.K.); tomas.strharsky01@upol.cz (T.S.); michnova.hana@gmail.com (H.M.); jan.hosek@upol.cz (J.H.)

[2] Department of Chemistry, University of Silesia, Szkolna 9, 40007 Katowice, Poland; violetta.kozik@us.edu.pl (V.K.); aswietlicka@us.edu.pl (A.Sw.)

[3] Department of Analytical Chemistry, Faculty of Natural Sciences, Comenius University, Ilkovicova 6, 84215 Bratislava, Slovakia; timotej.jankech@gmail.com (T.J.); milan.hutta@uniba.sk (M.H.)

[4] Central Mining Institute, Pl. Gwarkow 1, 40166 Katowice, Poland; smolin@gig.katowice.pl

[5] Global Change Research Institute CAS, Belidla 986/4a, 60300 Brno, Czech Republic; oravec.m@czechglobe.cz

[6] Faculty of Pharmacy, Comenius University, Odbojarov 10, 83232 Bratislava, Slovakia; devinsky@fpharm.uniba.sk

* Correspondence: andrzej.bak@us.edu.pl (A.B.); josef.jampilek@gmail.com (J.J.)

† Preliminary results presented at the 23rd International Electronic Conference on Synthetic Organic Chemistry, 15 November–15 December 2019; Available online: https://ecsoc-23.sciforum.net/.

Table S1. Theoretically estimated partition coefficient calculated by set of alternative methods for anilides **1–20**.

No.	logP[a]	miLogP[b]	ClogP[c]	ClogP[d]	ClogP[e]	ClogP[f]	ClogP[g]	MlogP[h]	AlogP[i]	ClogP[j]	ClogP[k]	ClogP[l]	ClogP[m]	ClogP[n]
**1**	3.12	3.47	3.14	3.20	3.66	3.57	3.73	3.08	3.29	3.08	3.61	3.18	3.71	3.18
**2**	3.35	3.81	3.44	3.61	3.27	4.00	4.22	3.70	4.46	2.56	3.48	3.66	4.14	3.44
**3**	3.34	3.81	3.44	3.61	4.33	4.00	5.01	3.70	4.46	3.68	3.48	3.66	4.00	3.45
**4**	4.75	4.75	4.35	4.23	4.59	4.78	5.24	4.41	4.31	3.81	4.26	4.3	5.14	4.6
**5**	5.36	5.36	4.95	4.75	5.21	5.38	5.96	5.08	4.82	4.45	5.07	4.86	5.73	5.5
**6**	5.33	5.36	4.95	4.75	5.94	5.38	6.30	5.08	4.82	5.02	5.84	4.86	5.61	5.51
**7**	4.71	5.01	4.57	4.78	4.86	5.11	5.54	4.58	4.55	4.30	4.57	4.84	5.44	4.8
**8**	4.15	4.24	3.84	3.85	4.27	4.32	4.56	3.95	4.19	3.37	3.99	3.9	4.57	4.04
**9**	4.21	4.37	3.96	4.13	5.02	4.48	5.30	4.04	4.31	4.17	3.97	4.17	4.72	4.14
**10**	4.06	4.45	4.08	4.22	5.23	4.59	5.25	4.23	4.55	4.25	4.17	4.26	4.74	4.23
**11**	4.64	4.88	4.47	4.51	5.45	4.94	5.72	4.49	4.43	4.62	4.95	4.57	5.15	4.88
**12**	4.61	4.88	4.47	4.51	4.71	4.94	5.36	4.49	4.43	4.05	4.50	4.57	5.29	4.75
**13**	4.51	5.10	4.71	4.87	5.01	5.22	5.52	4.77	4.78	4.37	4.76	4.93	5.46	4.9
**14**	4.01	4.45	4.08	4.22	3.78	4.59	5.21	4.23	4.55	3.68	3.54	4.26	4.74	3.8
**15**	4.50	4.97	4.59	4.60	4.35	5.05	6.11	4.69	4.67	4.13	4.06	4.66	5.31	4.84
**16**	4.45	5.10	4.71	4.87	4.50	5.22	5.78	4.77	4.78	4.37	4.13	4.93	5.46	4.86
**17**	3.76	4.25	3.06	2.10	3.55	4.39	5.48	3.92	4.10	4.02	3.27	4.25	4.34	4.16
**18**	4.10	4.25	3.06	2.10	4.99	4.39	5.60	3.92	4.10	4.58	4.39	4.25	4.34	4.27
**19**	5.16	5.84	5.43	5.66	5.08	5.99	5.45	5.52	5.39	4.70	5.45	5.76	6.32	5.32
**20**	5.22	5.71	5.29	5.44	4.74	5.85	6.35	5.45	5.17	4.58	5.29	5.55	6.16	5.67

[a] clogPS, [b] Molinspirations, [c] OSIRIS property explorer, [d] HyperChem 7.0, [e] Sybyl-X, [f] Marvin Sketch (ChemAxon) 15, [g] ChemSketch 2015, [h] Dragon 6.0, [i] Dragon 6.0, [j] Kowwin, [k] XlogP3, [l] ChemDraw, [m] ChemBioDraw, [n] ACD/Percepta.

Table S2. Theoretically estimated pK_a calculated by ACD/Percepta/pKa for anilides **1–20**.

No.	pK_a[a]	pK_a[b]
**1**	13.98	0.42
**2**	10.79	0.04
**3**	11.38	-0.95
**4**	12.17	-1.12
**5**	11.50	-1.92
**6**	11.76	-1.80
**7**	12.17	-1.32
**8**	12.22	-0.62
**9**	12.32	-1.13
**10**	12.47	-2.44
**11**	12.43	-1.20
**12**	12.17	-1.12
**13**	12.50	-1.43
**14**	12.19	-1.52
**15**	12.34	-2.24
**16**	12.34	-2.44
**17**	12.00	-5.76
**18**	12.22	-4.65
**19**	11.40	-3.54
**20**	10.42	-2.12

[a] acid, [b] base

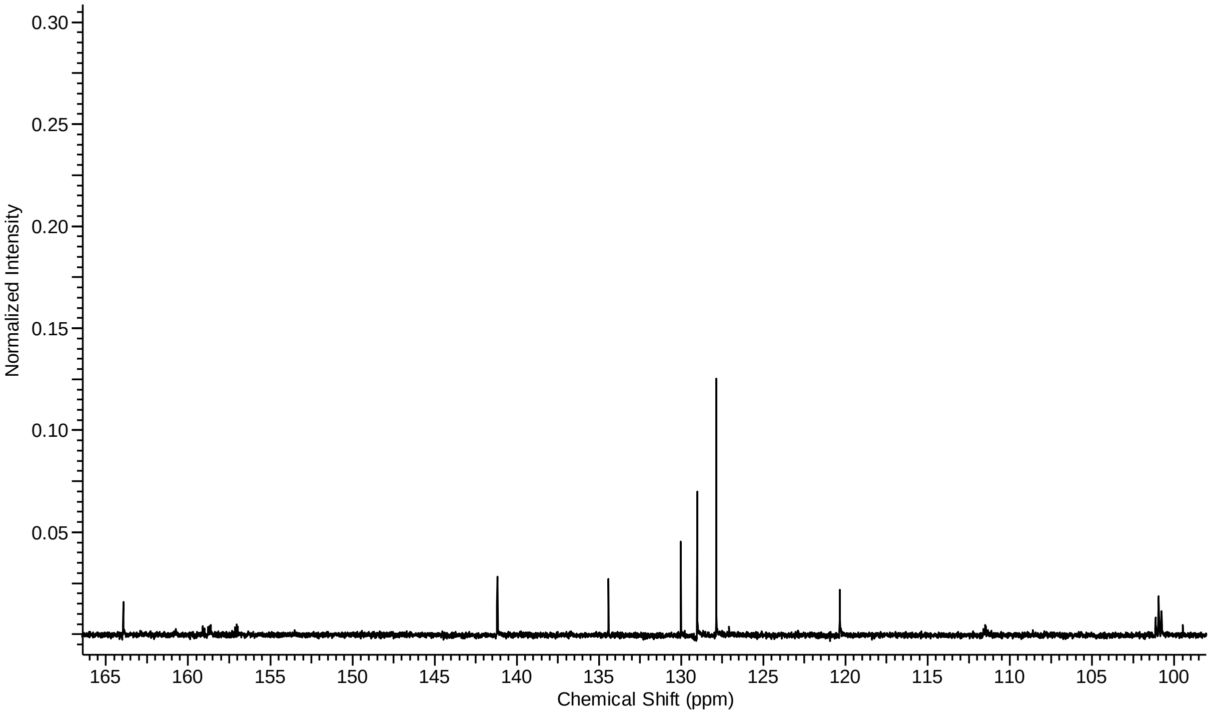

Figure S1. ^{13}C-NMR (DMSO-d_6) spectrum of (2*E*)-3-phenyl-*N*-(2,4,6-trifluorophenyl)prop-2-enamide (**2**).

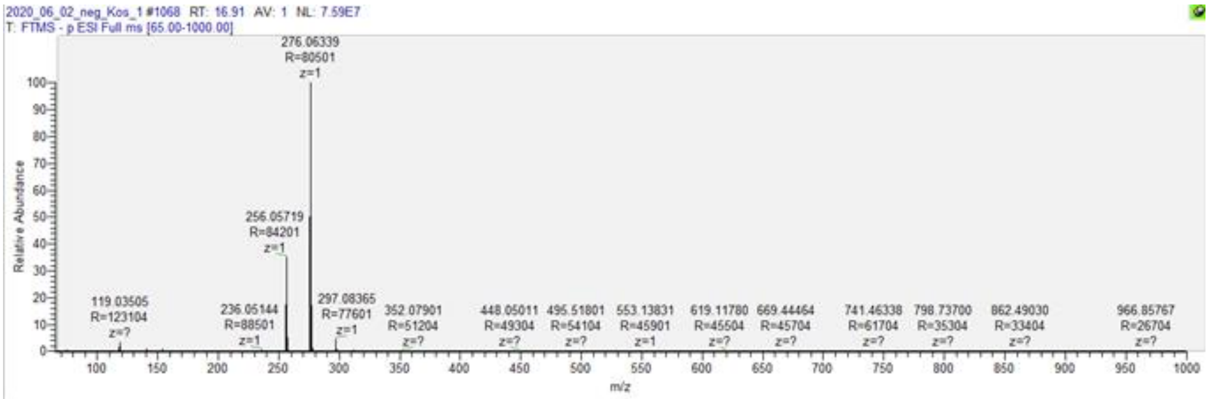

Figure S2. HR-MS record of (2*E*)-3-phenyl-*N*-(2,4,6-trifluorophenyl)prop-2-enamide (**2**).

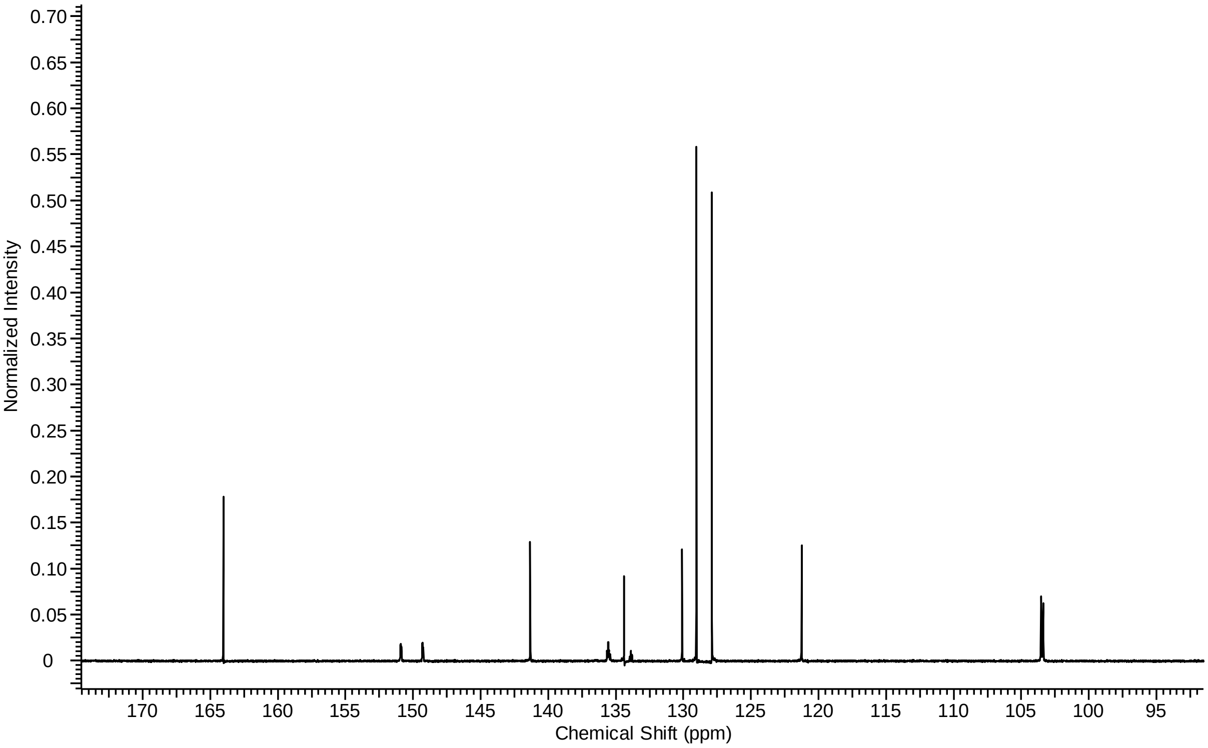

Figure S3. ^{13}C-NMR (DMSO-d_6) spectrum of (2*E*)-3-phenyl-*N*-(3,4,5-trifluorophenyl)prop-2-enamide (**3**).

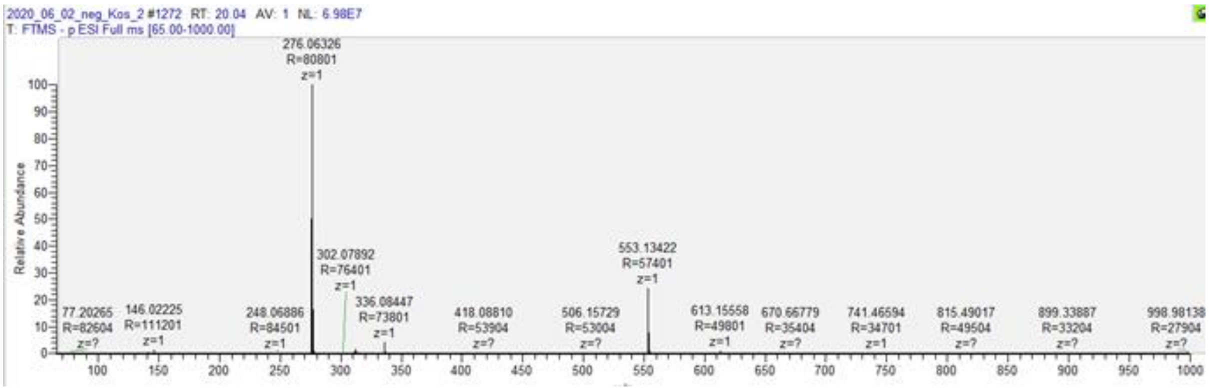

Figure S4. HR-MS record of (2*E*)-3-phenyl-*N*-(3,4,5-trifluorophenyl)prop-2-enamide (**3**).

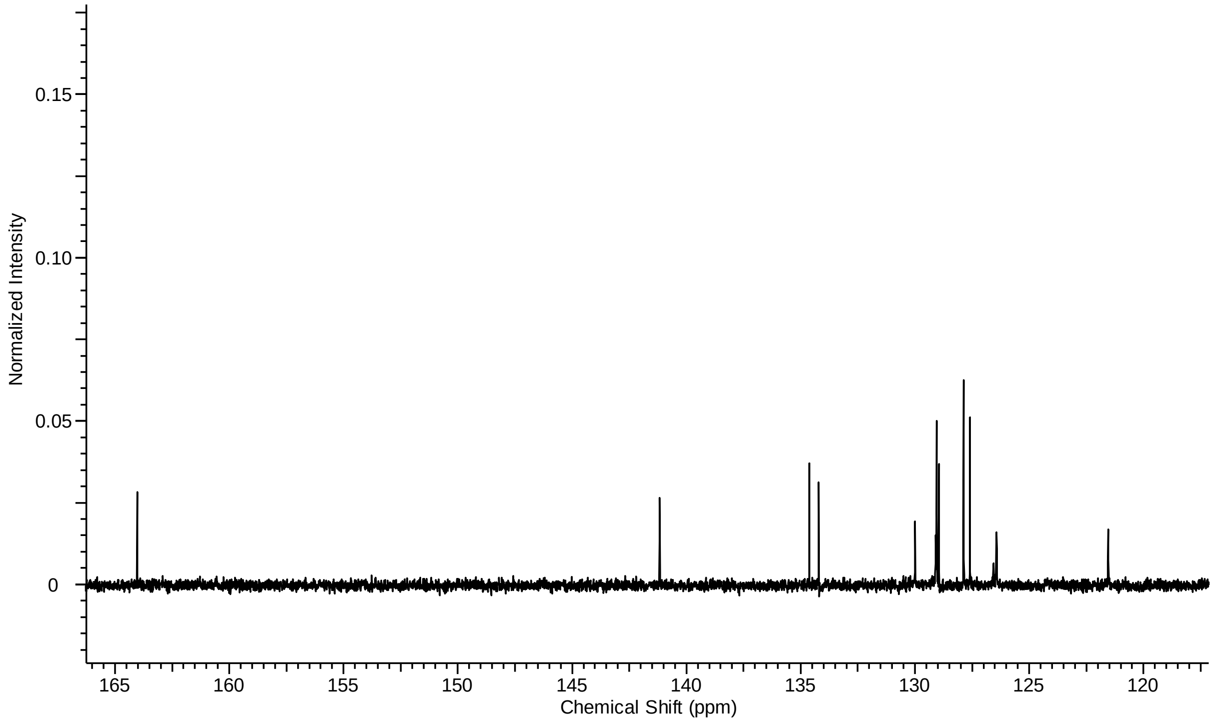

Figure S5. ^{13}C-NMR (DMSO-d_6) spectrum of (2*E*)-*N*-(2,4-dichlorophenyl)-3-phenylprop-2-enamide (**4**).

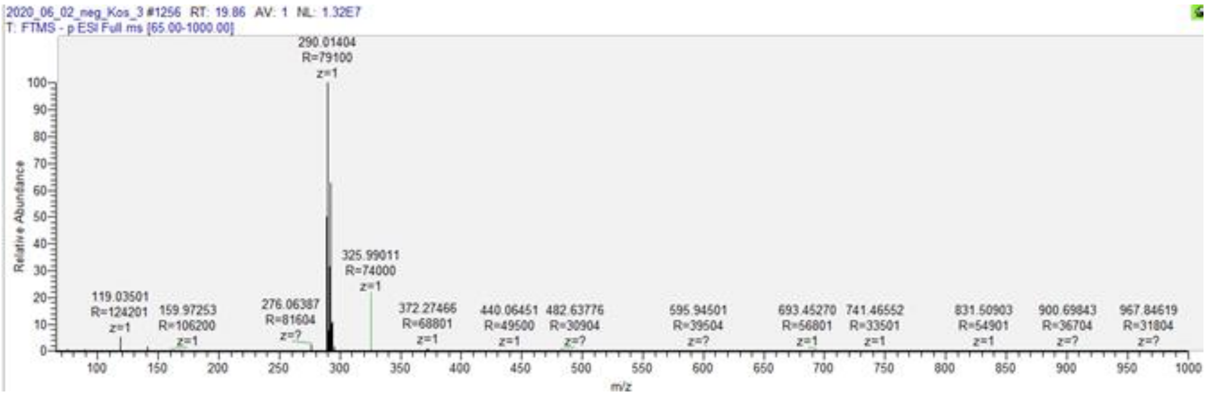

Figure S6. HR-MS record of (2*E*)-*N*-(2,4-dichlorophenyl)-3-phenylprop-2-enamide (**4**).

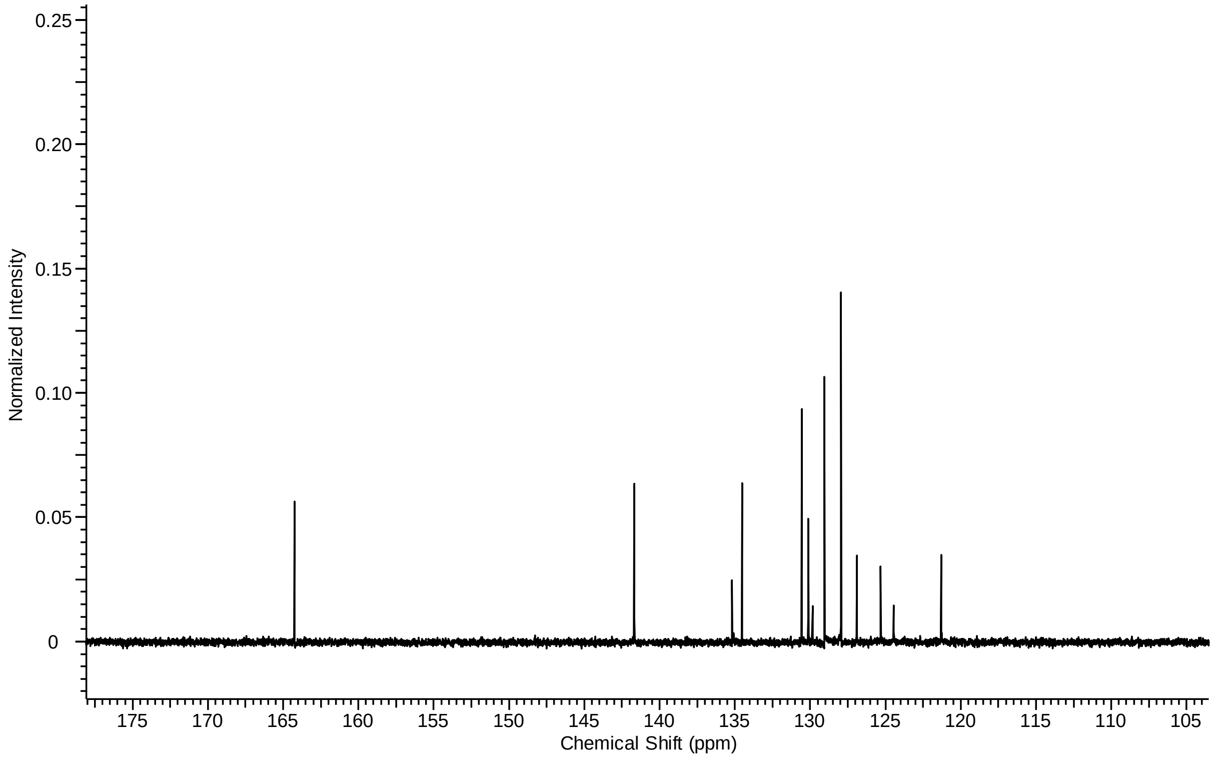

Figure S7. ^{13}C-NMR (DMSO-d_6) spectrum of (2*E*)-3-phenyl-*N*-(2,4,5-trichlorophenyl)prop-2-enamide (**5**).

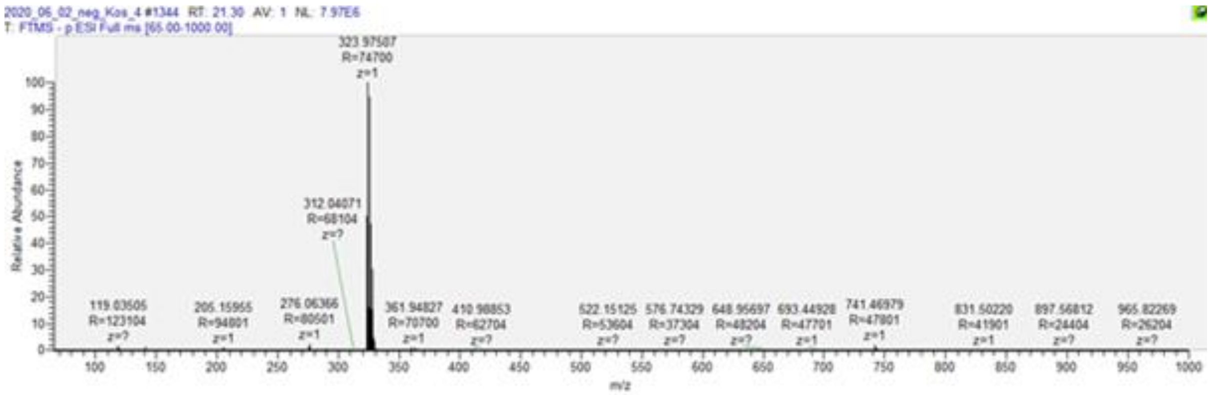

Figure S8. HR-MS record of (2*E*)-3-phenyl-*N*-(2,4,5-trichlorophenyl)prop-2-enamide (**5**).

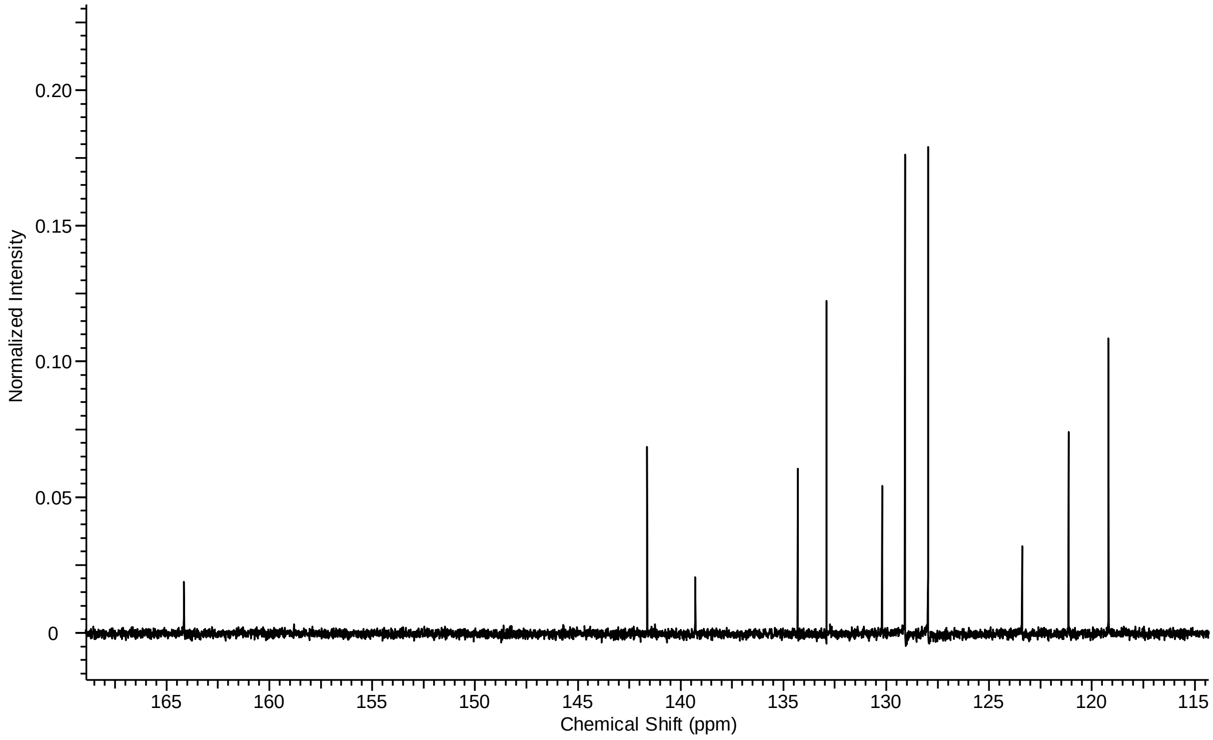

Figure S9. ^{13}C-NMR (DMSO-d_6) spectrum of (2*E*)-3-phenyl-*N*-(3,4,5-trichlorophenyl)prop-2-enamide (**6**).

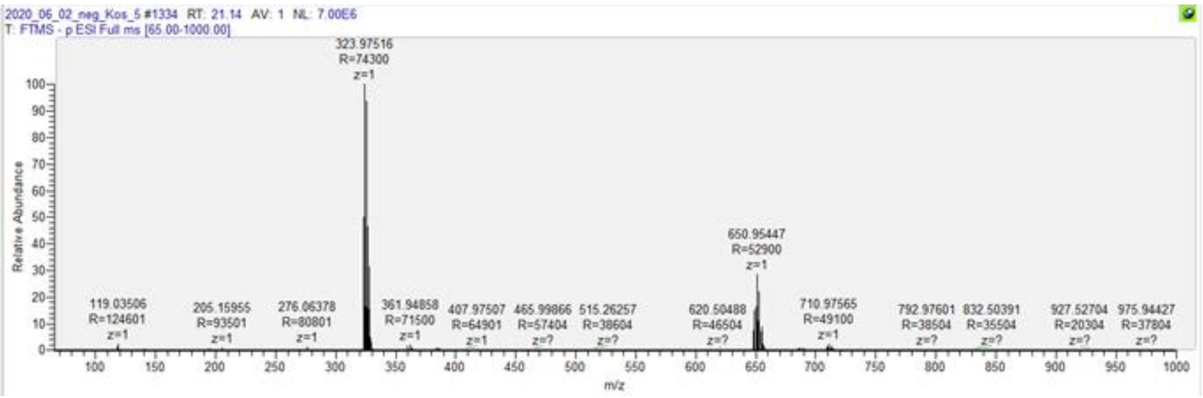

Figure S10. HR-MS record of (2*E*)-3-phenyl-*N*-(3,4,5-trichlorophenyl)prop-2-enamide (**6**).

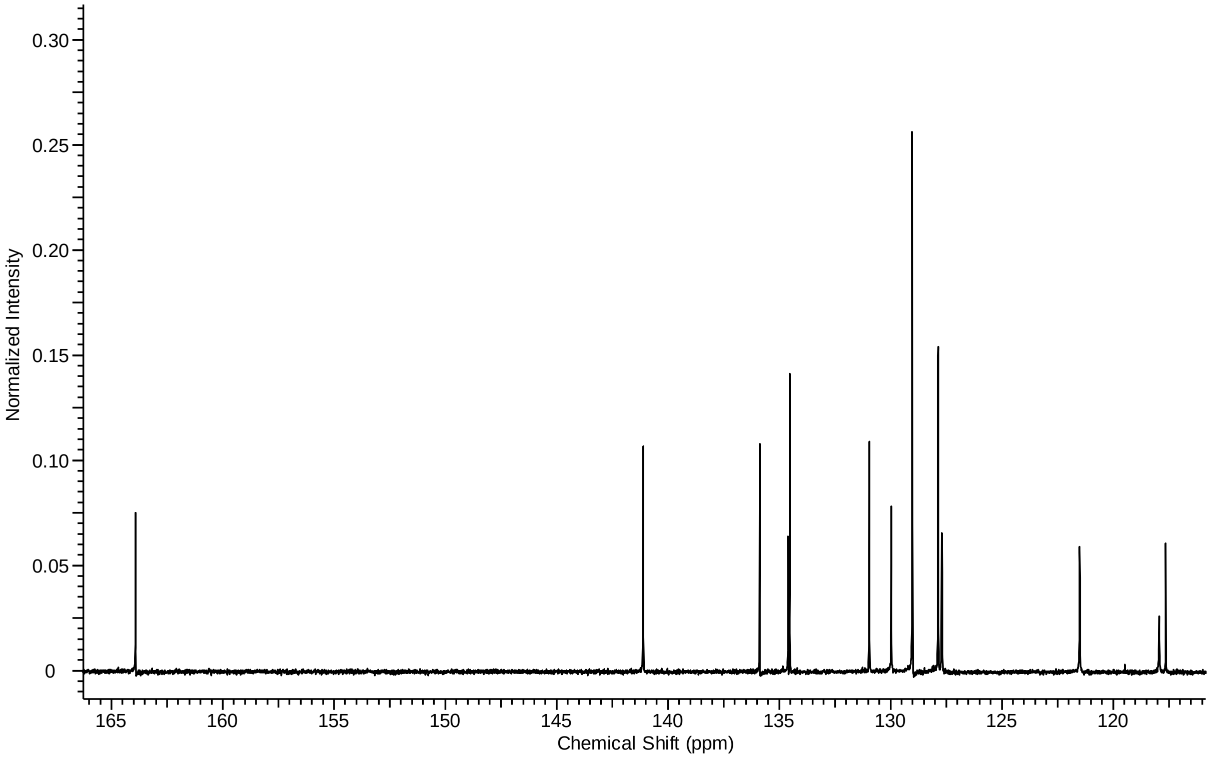

Figure S11. ^{13}C-NMR (DMSO-d_6) spectrum of (2*E*)-*N*-(2,4-dibromophenyl)-3-phenylprop-2-enamide (**7**).

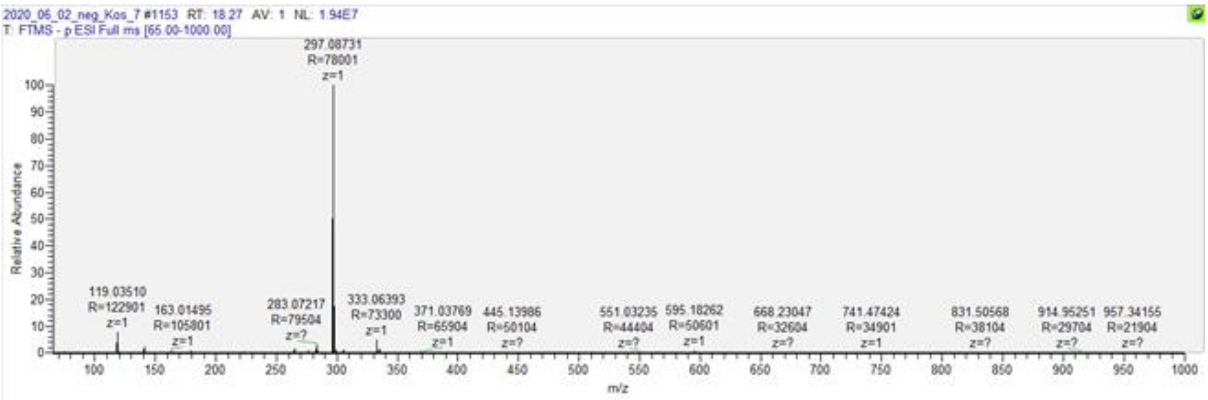

Figure S12. HR-MS record of (2*E*)-*N*-(2,4-dibromophenyl)-3-phenylprop-2-enamide (**7**).

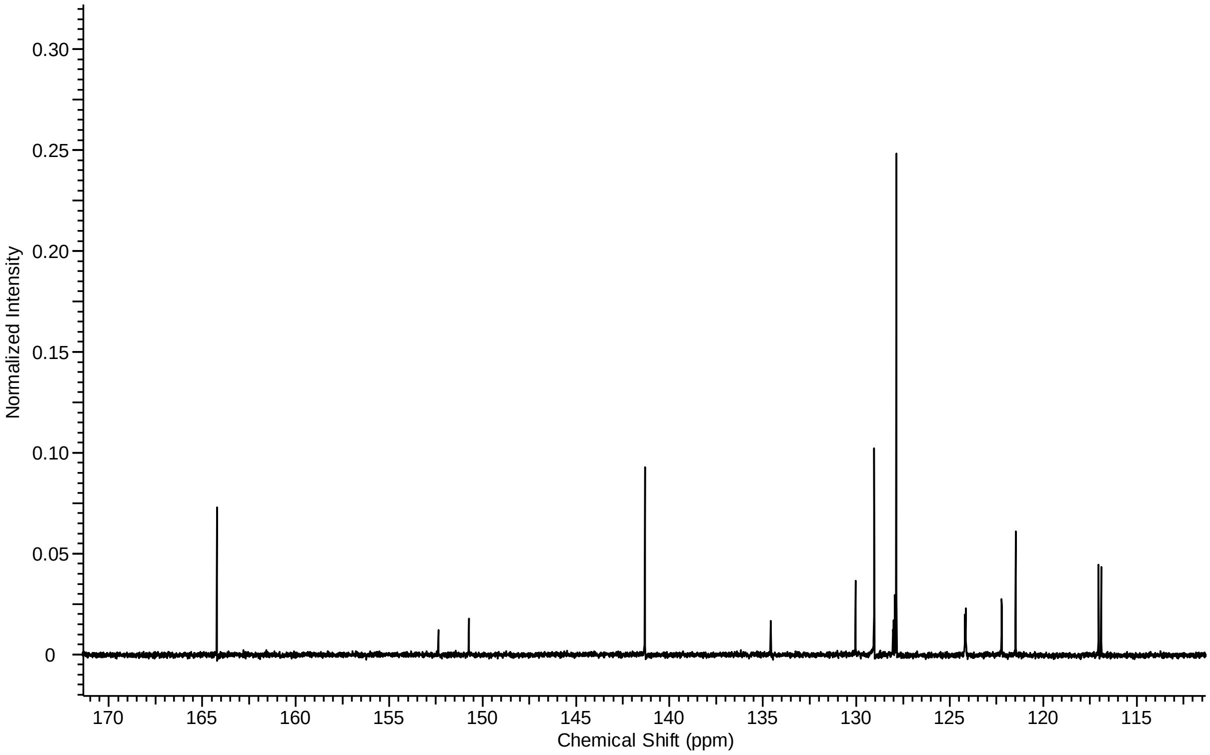

Figure S13. ^{13}C-NMR (DMSO-d_6) spectrum of (2*E*)-*N*-(5-chloro-2-fluorophenyl)-3-phenylprop-2-enamide (**8**).

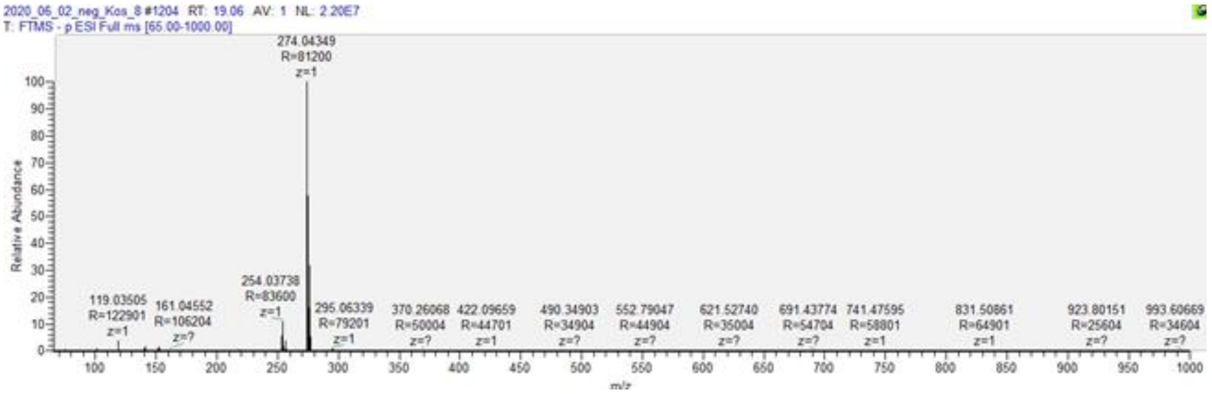

Figure S14. HR-MS record of (2*E*)-*N*-(5-chloro-2-fluorophenyl)-3-phenylprop-2-enamide (**8**).

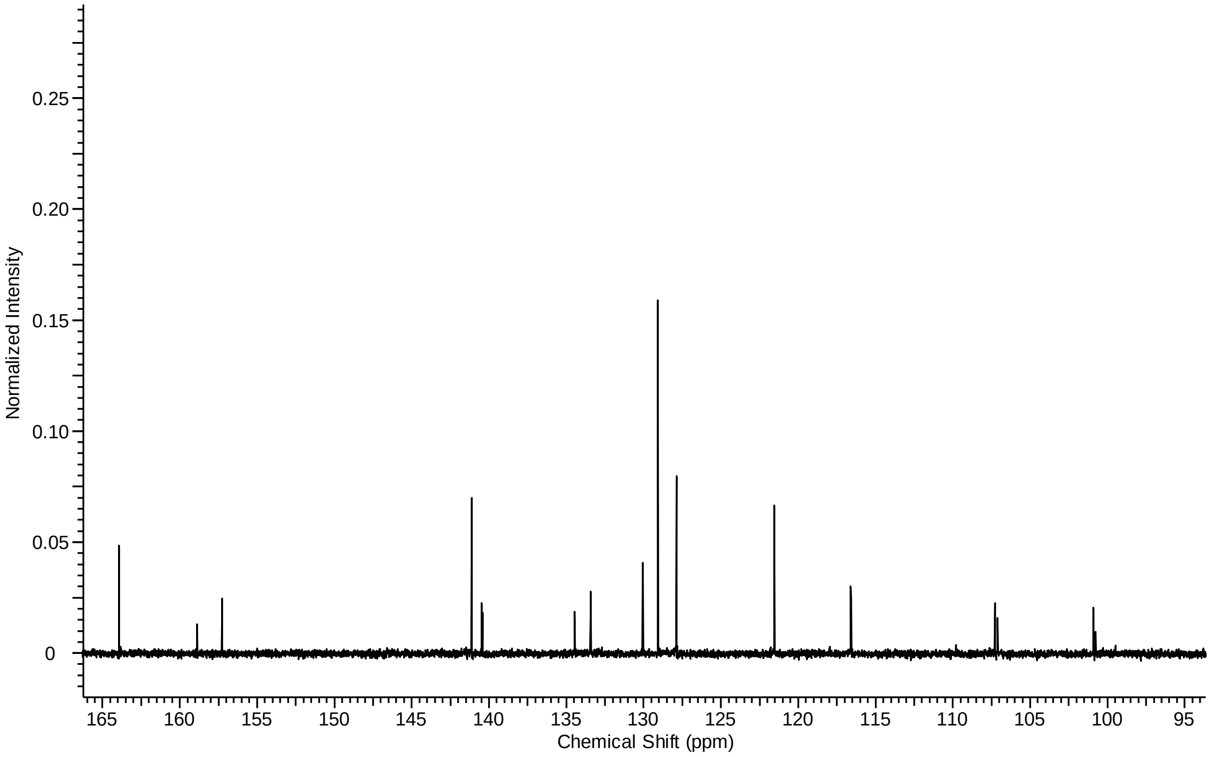

Figure S15. ^{13}C-NMR (DMSO-d_6) spectrum of (2*E*)-*N*-(4-bromo-3-fluorophenyl)-3-phenylprop-2-enamide (**9**).

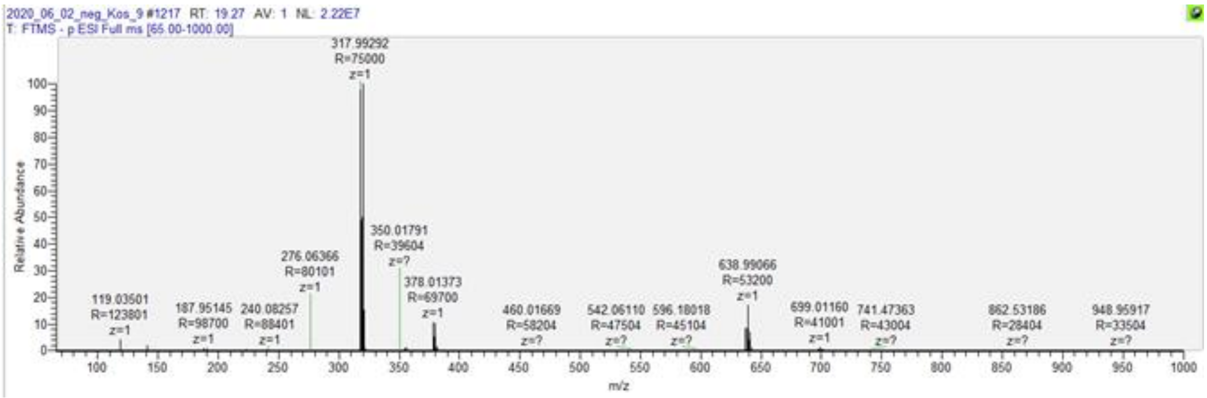

Figure S16. HR-MS record of (2*E*)-*N*-(4-bromo-3-fluorophenyl)-3-phenylprop-2-enamide (**9**).

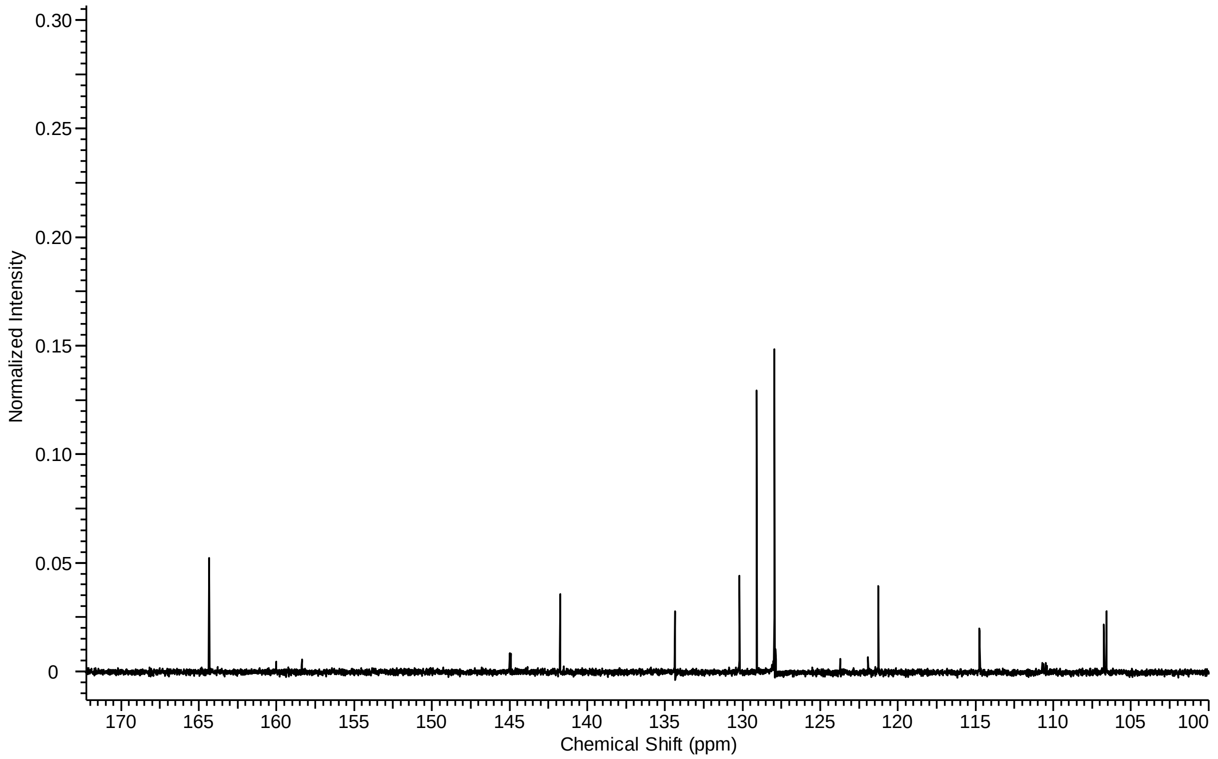

Figure S17. ^{13}C-NMR (DMSO-d_6) spectrum of (2*E*)-*N*-[3-fluoro-4-(trifluoromethyl)phenyl]-3-phenylprop-2-enamide (**10**).

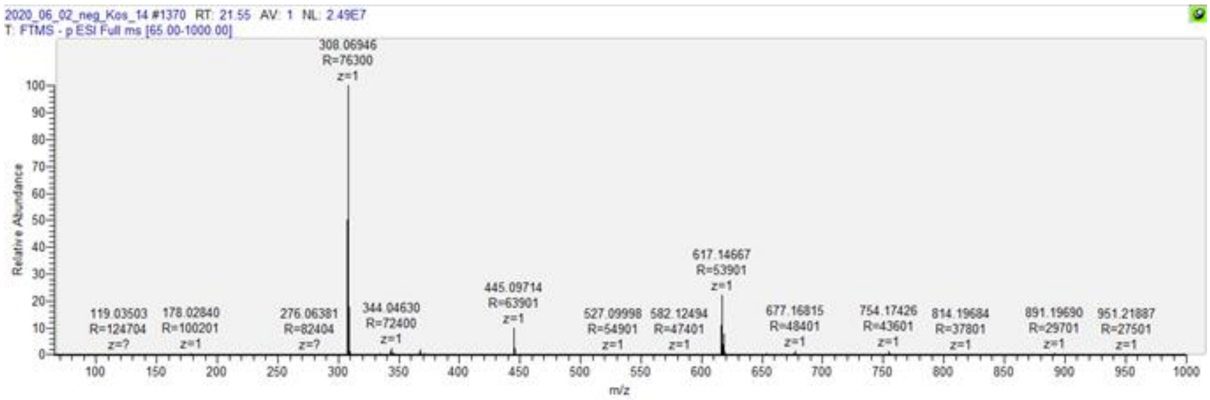

Figure S18. HR-MS record of (2*E*)-*N*-[3-fluoro-4-(trifluoromethyl)phenyl]-3-phenylprop-2-enamide (**10**).

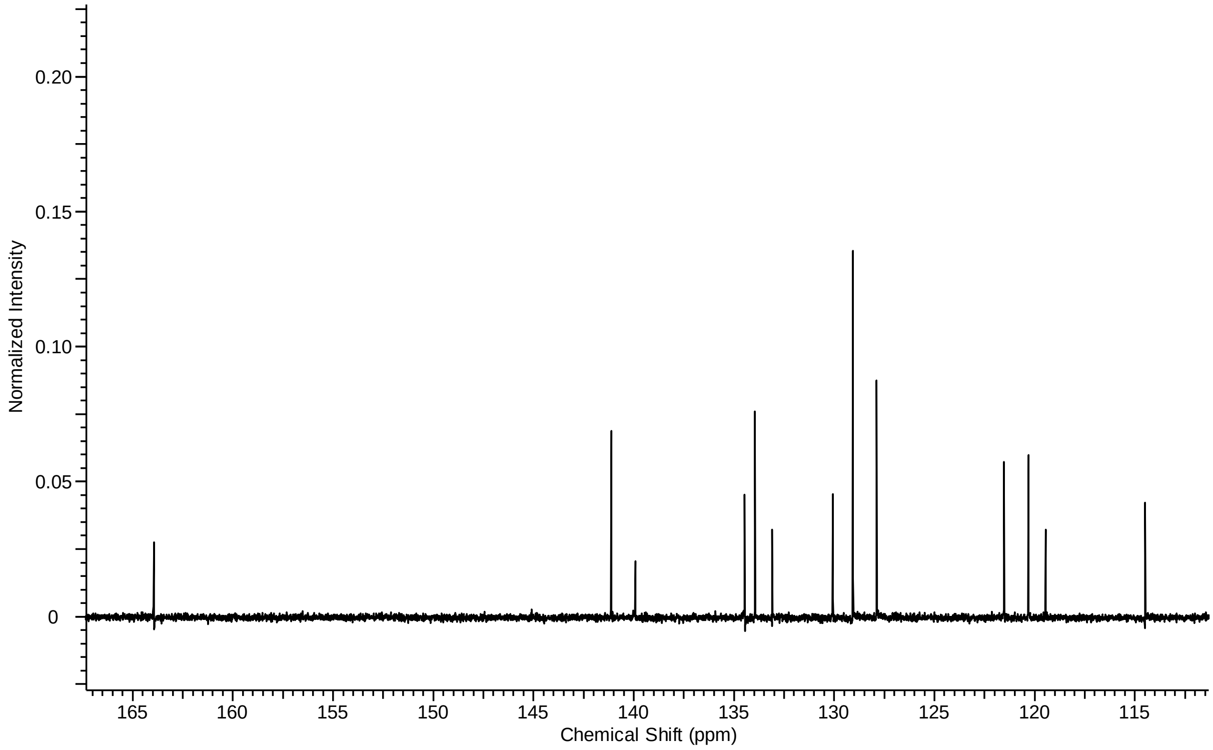

Figure S19. ${}^{13}C$-NMR (DMSO-d_6) spectrum of (2*E*)-*N*-(4-bromo-3-chlorophenyl)-3-phenylprop-2-enamide (**11**).

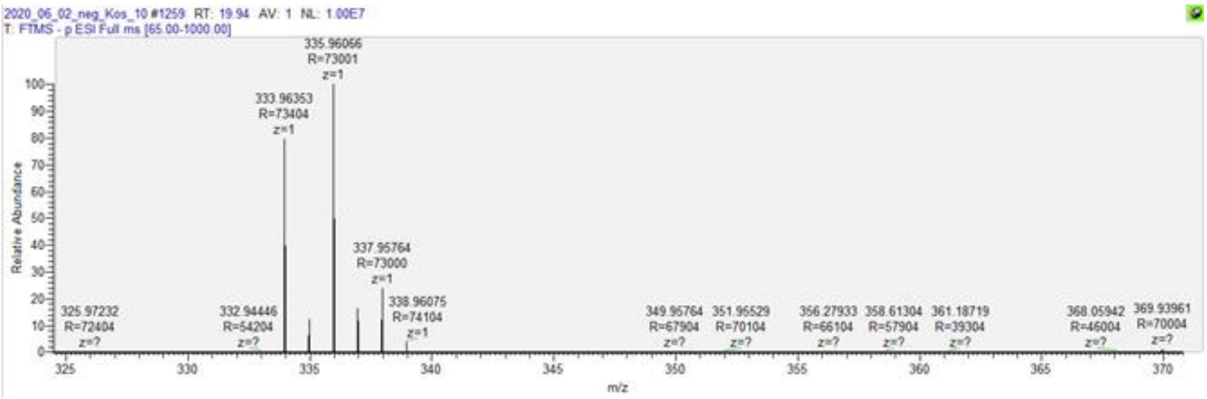

Figure S20. HR-MS record of (2*E*)-*N*-(4-bromo-3-chlorophenyl)-3-phenylprop-2-enamide (**11**).

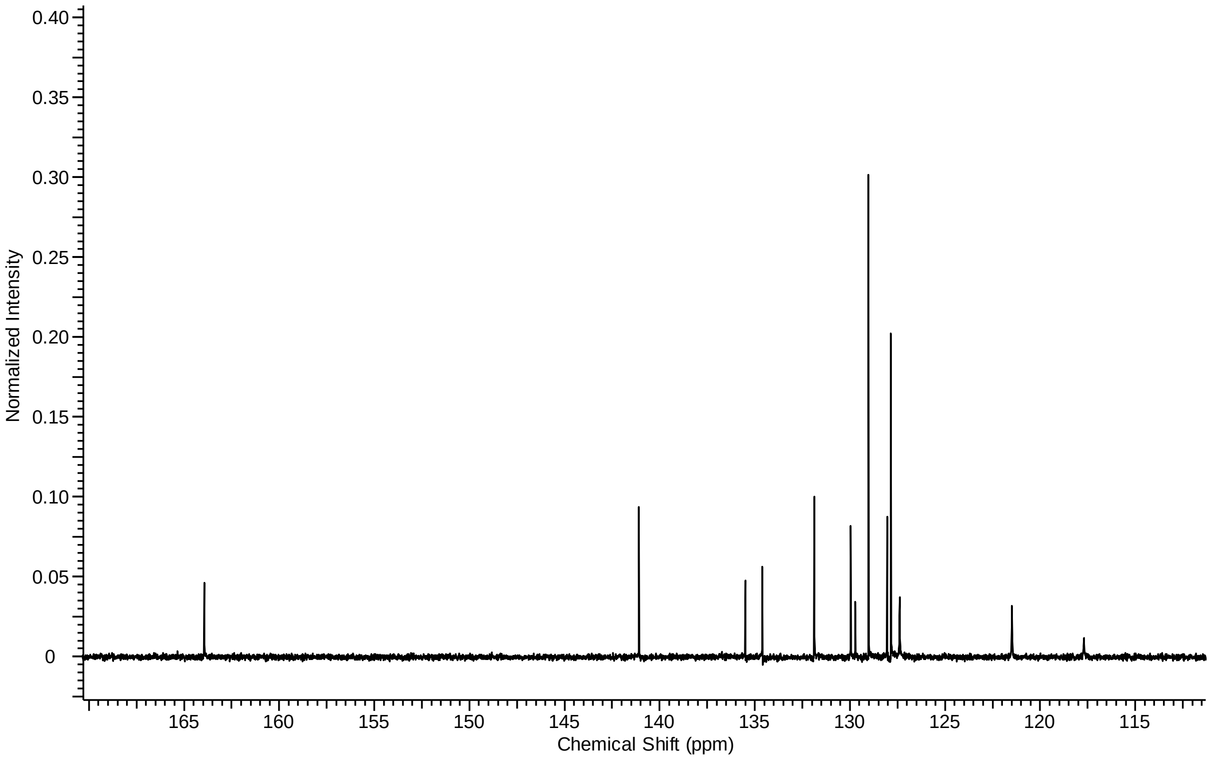

Figure S21. ^{13}C-NMR (DMSO-d_6) spectrum of (2*E*)-*N*-(2-bromo-4-chlorophenyl)-3-phenylprop-2-enamide (**12**).

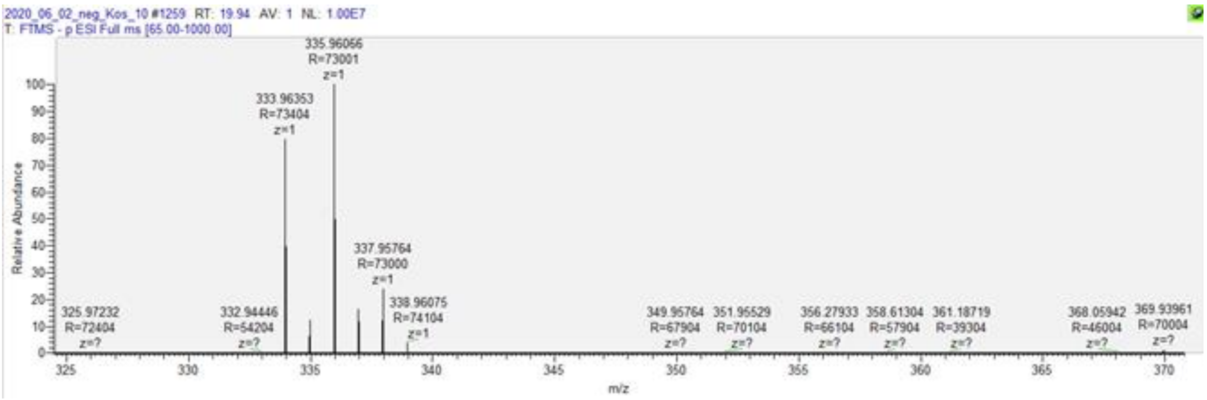

Figure S22. HR-MS record of (2*E*)-*N*-(2-bromo-4-chlorophenyl)-3-phenylprop-2-enamide (**12**).

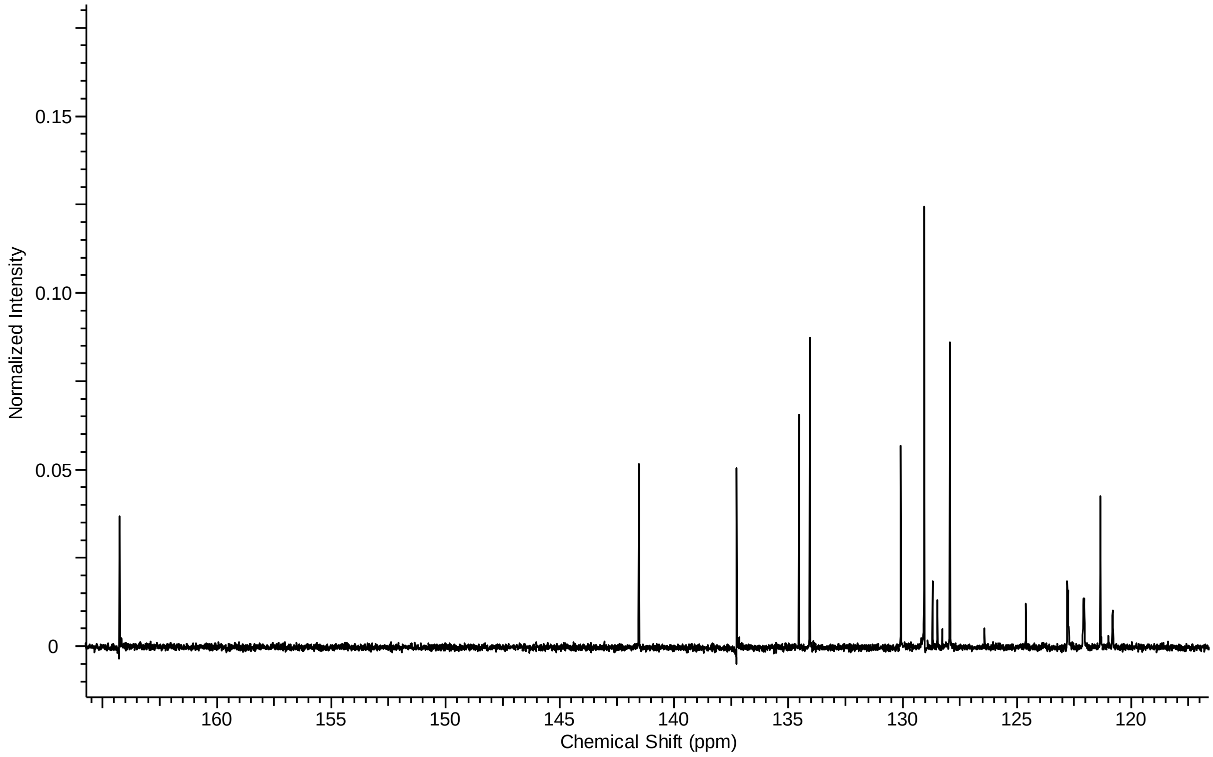

Figure S23. ^{13}C-NMR (DMSO-d_6) spectrum of (2*E*)-*N*-[2-bromo-5-(trifluoromethyl)phenyl]-3-phenylprop-2-enamide (**13**).

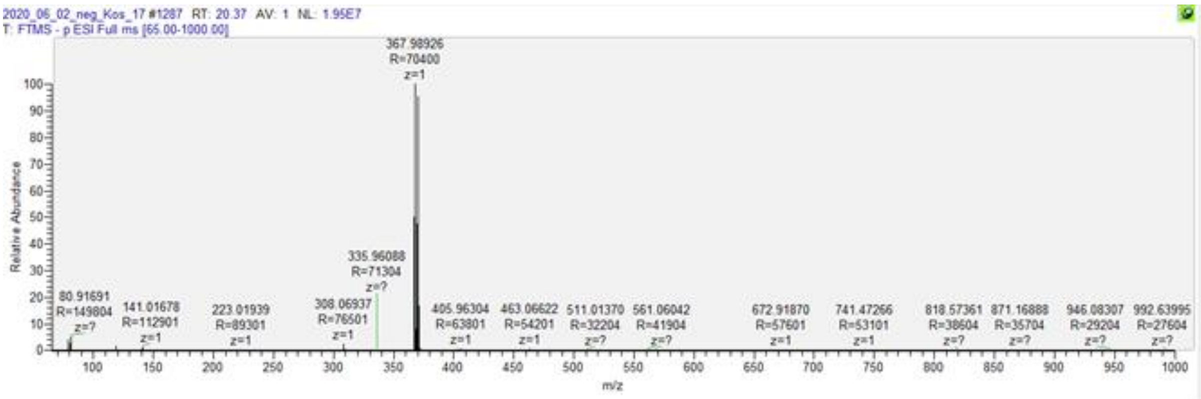

Figure S24. HR-MS record of (2*E*)-*N*-[2-bromo-5-(trifluoromethyl)phenyl]-3-phenylprop-2-enamide (**13**).

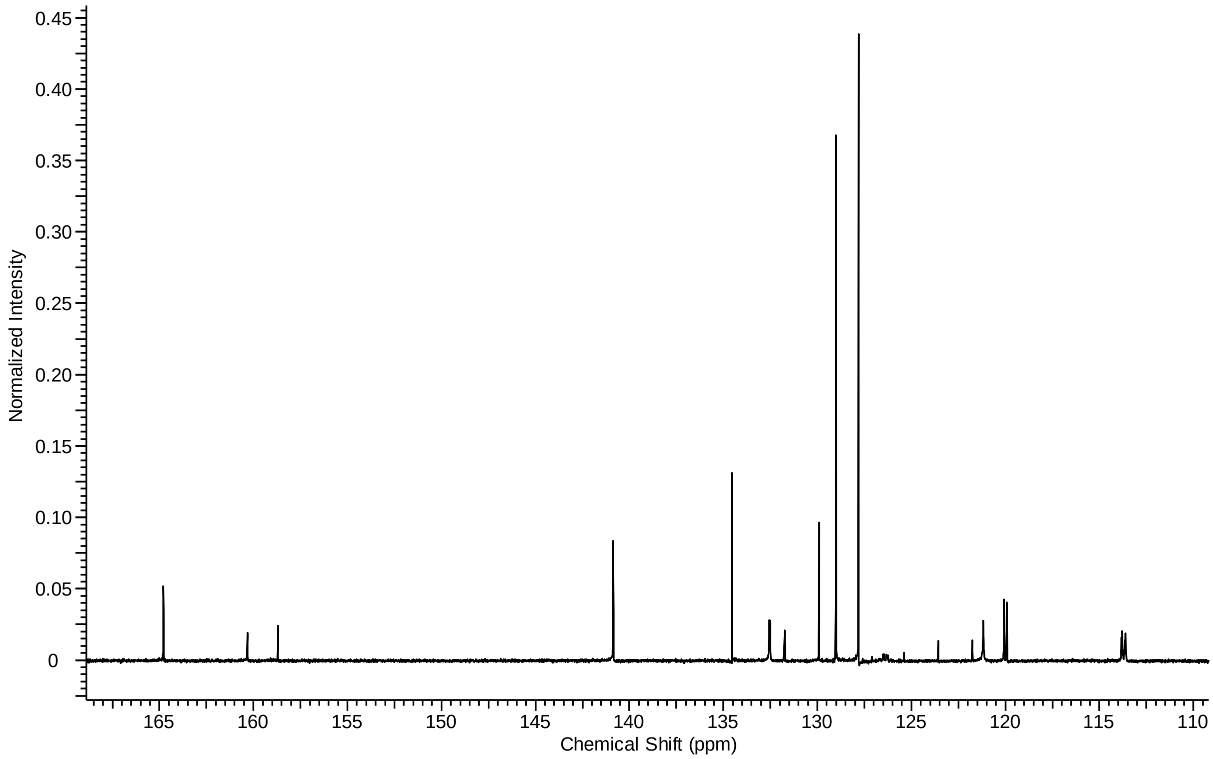

Figure S25. ^{13}C-NMR (DMSO-d_6) spectrum of (2*E*)-*N*-[4-fluoro-2-(trifluoromethyl)phenyl]-3-phenylprop-2-enamide (**14**).

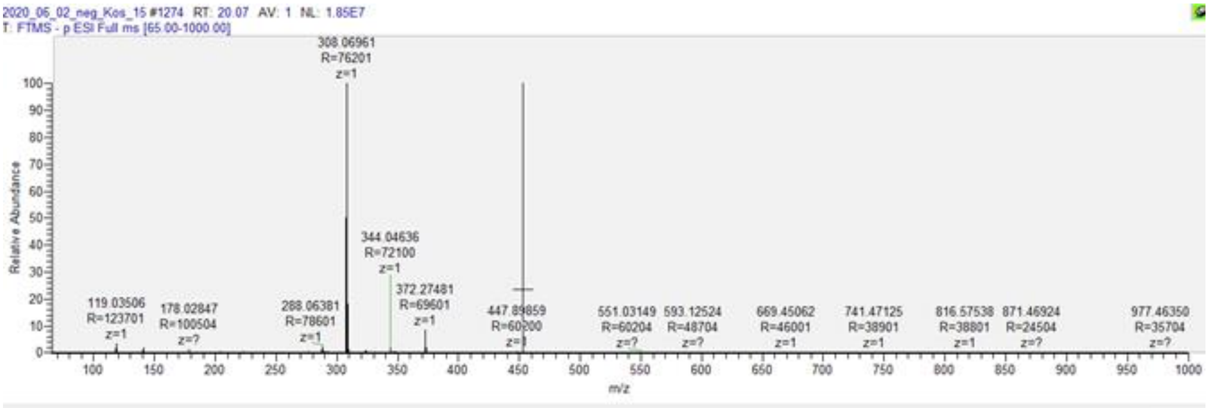

Figure S26. HR-MS record of (2*E*)-*N*-[4-fluoro-2-(trifluoromethyl)phenyl]-3-phenylprop-2-enamide (**14**).

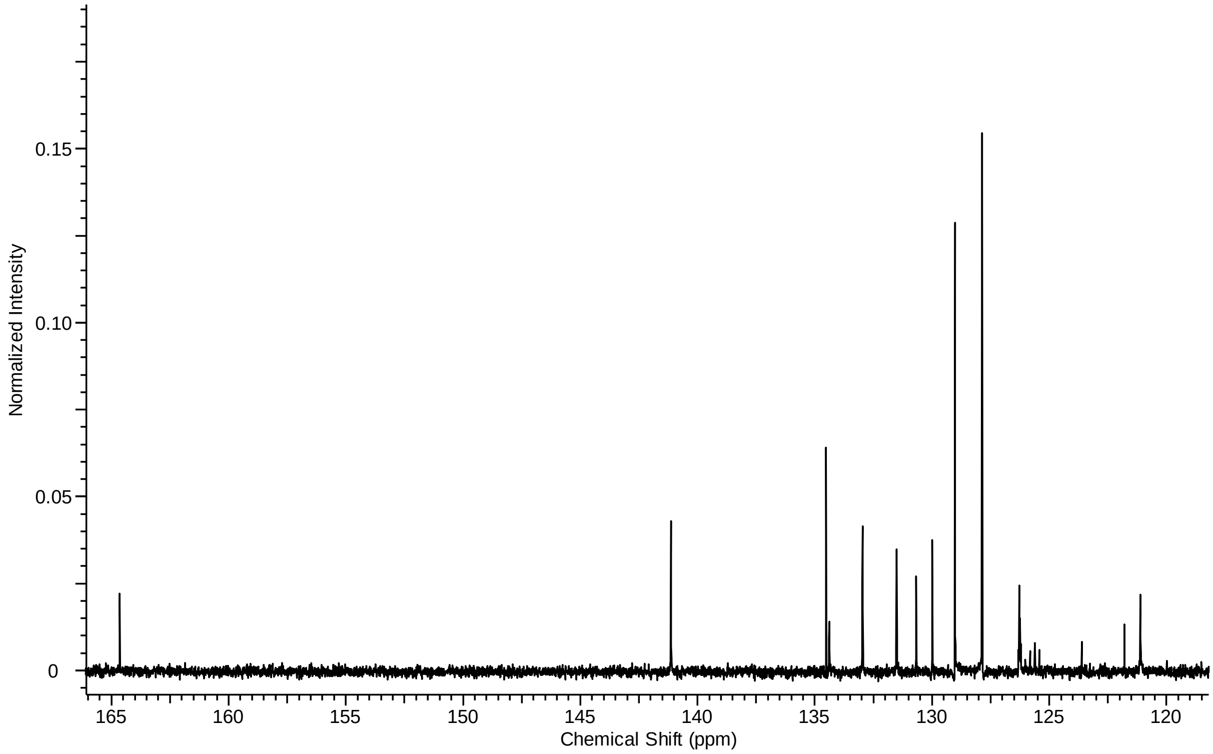

Figure S27. ^{13}C-NMR (DMSO-d_6) spectrum of (2*E*)-*N*-[4-chloro-2-(trifluoromethyl)phenyl]-3-phenylprop-2-enamide (**15**).

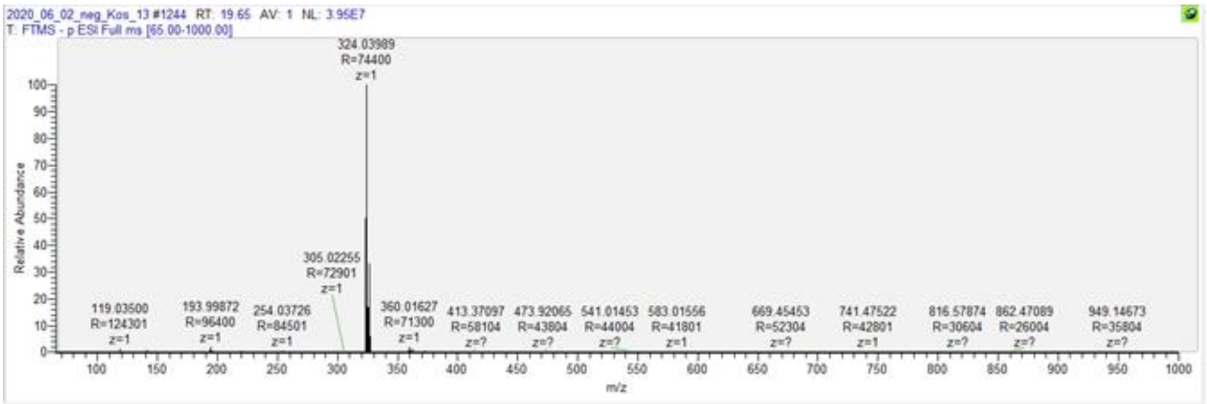

Figure S28. HR-MS record of (2*E*)-*N*-[4-chloro-2-(trifluoromethyl)phenyl]-3-phenylprop-2-enamide (**15**).

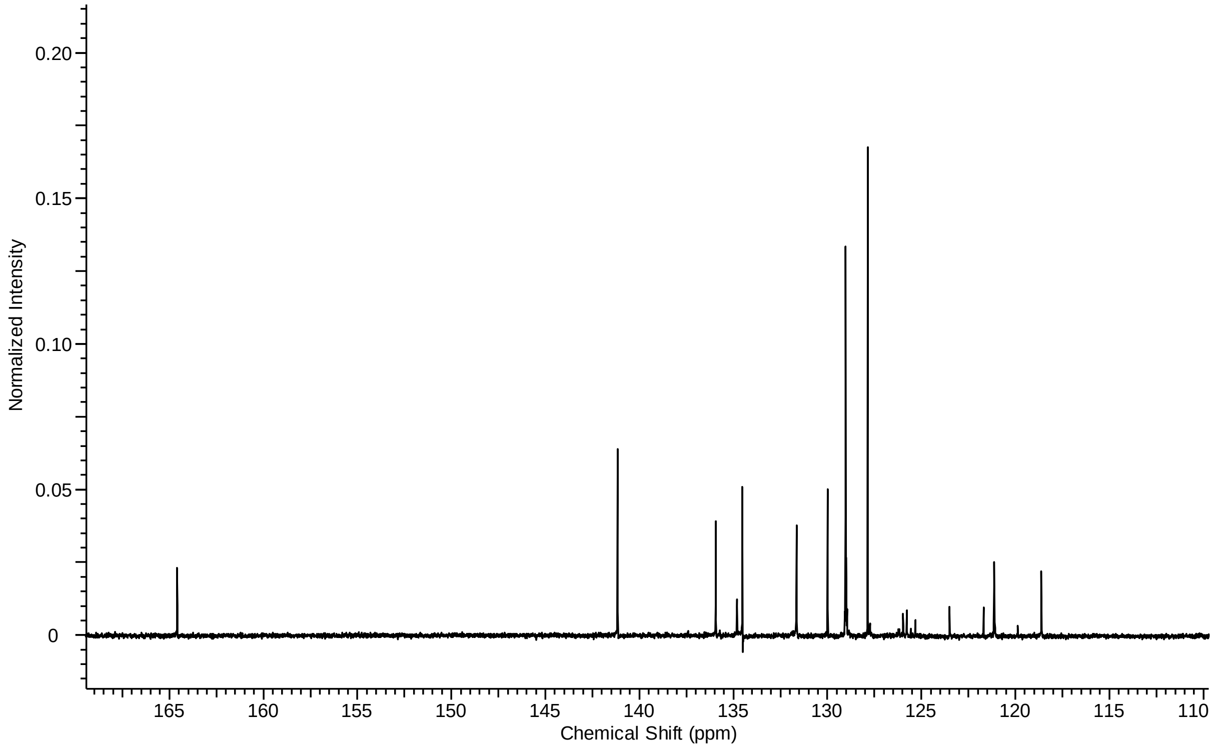

Figure S29. ^{13}C-NMR (DMSO-d_6) spectrum of (2*E*)-*N*-[4-bromo-2-(trifluoromethyl)phenyl]-3-phenylprop-2-enamide (**16**).

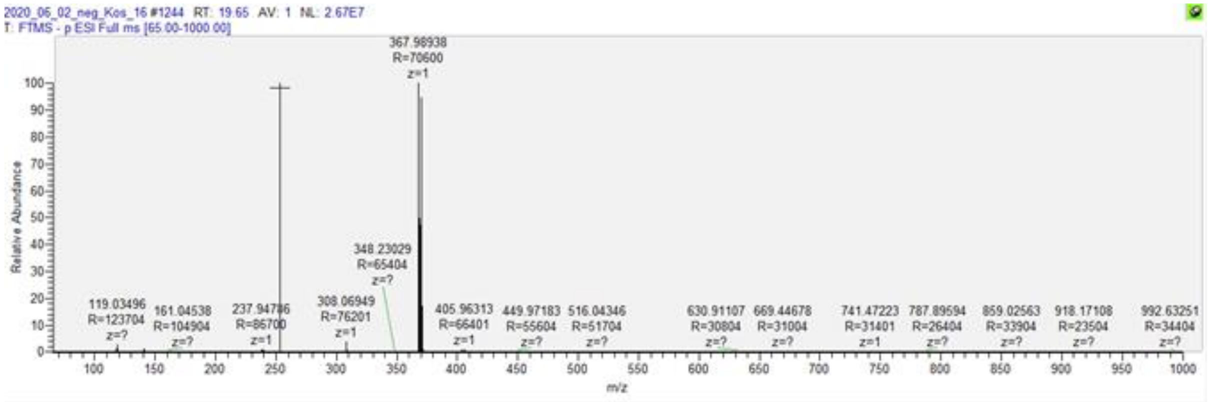

Figure S30. HR-MS record of (2*E*)-*N*-[4-bromo-2-(trifluoromethyl)phenyl]-3-phenylprop-2-enamide (**16**).

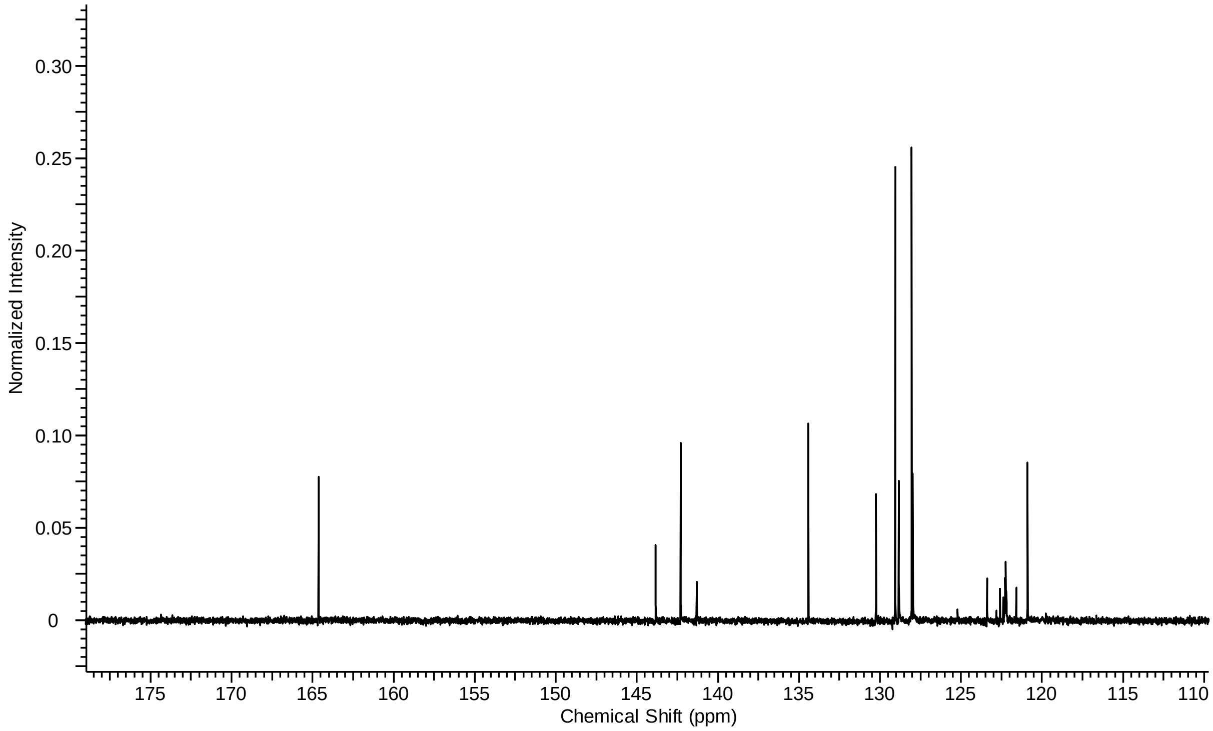

Figure S31. ^{13}C-NMR (DMSO-d_6) spectrum of (2*E*)-*N*-[4-nitro-2-(trifluoromethyl)phenyl]-3-phenylprop-2-enamide (**17**).

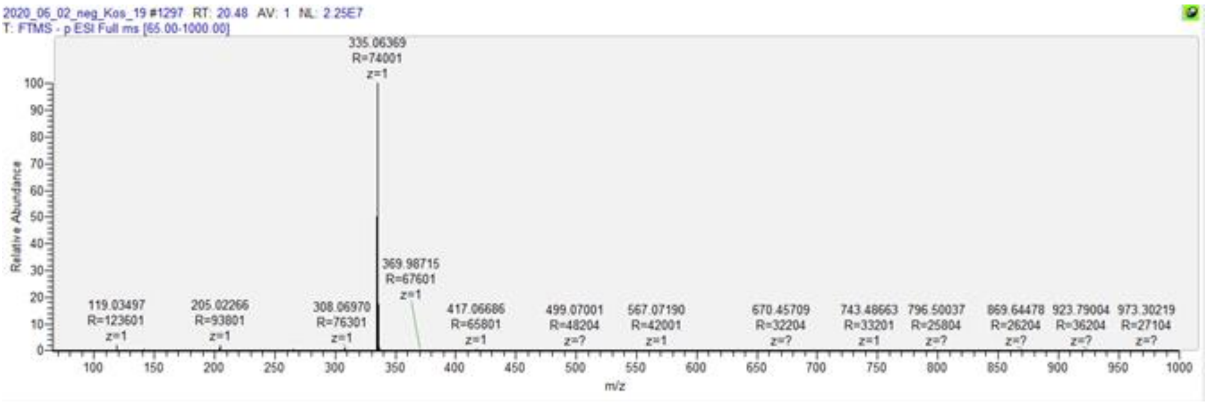

Figure S32. HR-MS record of (2*E*)-*N*-[4-nitro-2-(trifluoromethyl)phenyl]-3-phenylprop-2-enamide (**17**).

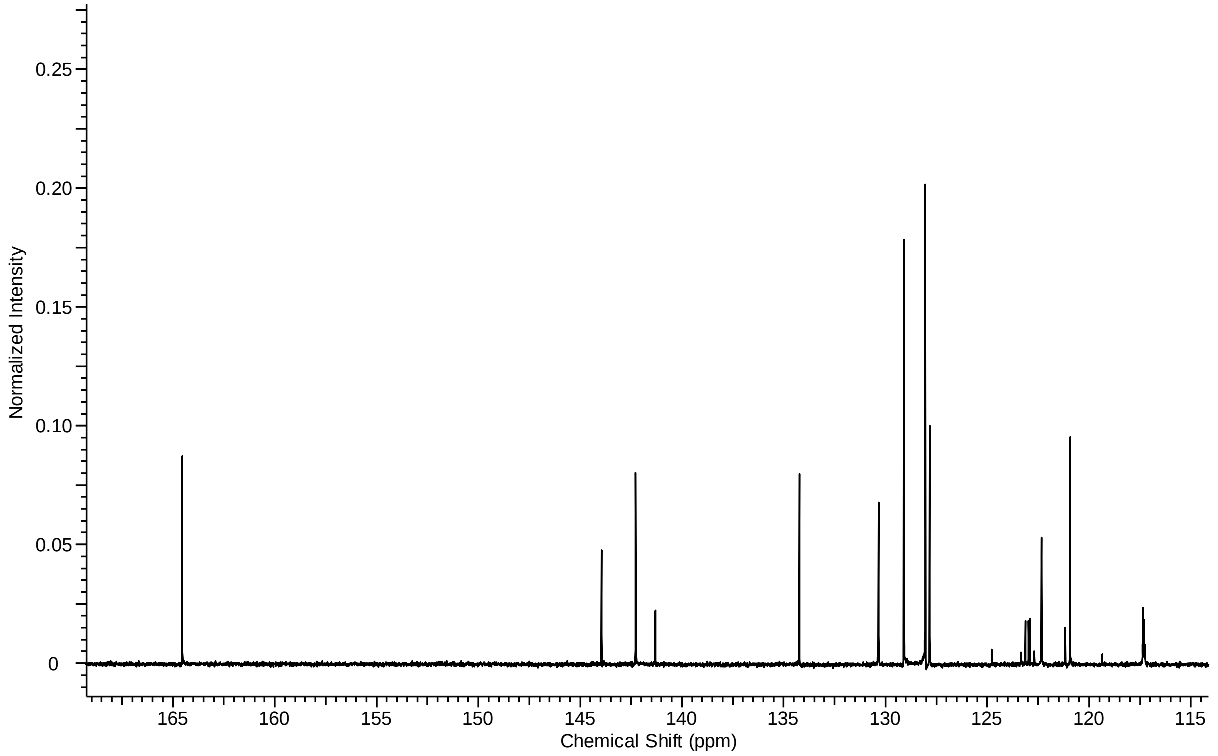

Figure S33. ^{13}C-NMR (DMSO-d_6) spectrum of (2*E*)-*N*-[4-nitro-3-(trifluoromethyl)phenyl]-3-phenylprop-2-enamide (**18**).

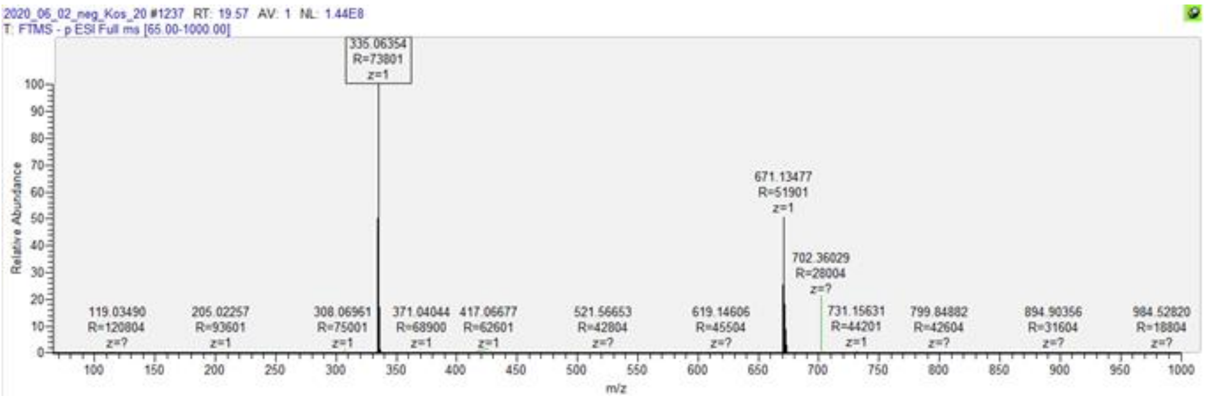

Figure S34. HR-MS record of (2*E*)-*N*-[4-nitro-3-(trifluoromethyl)phenyl]-3-phenylprop-2-enamide (**18**).

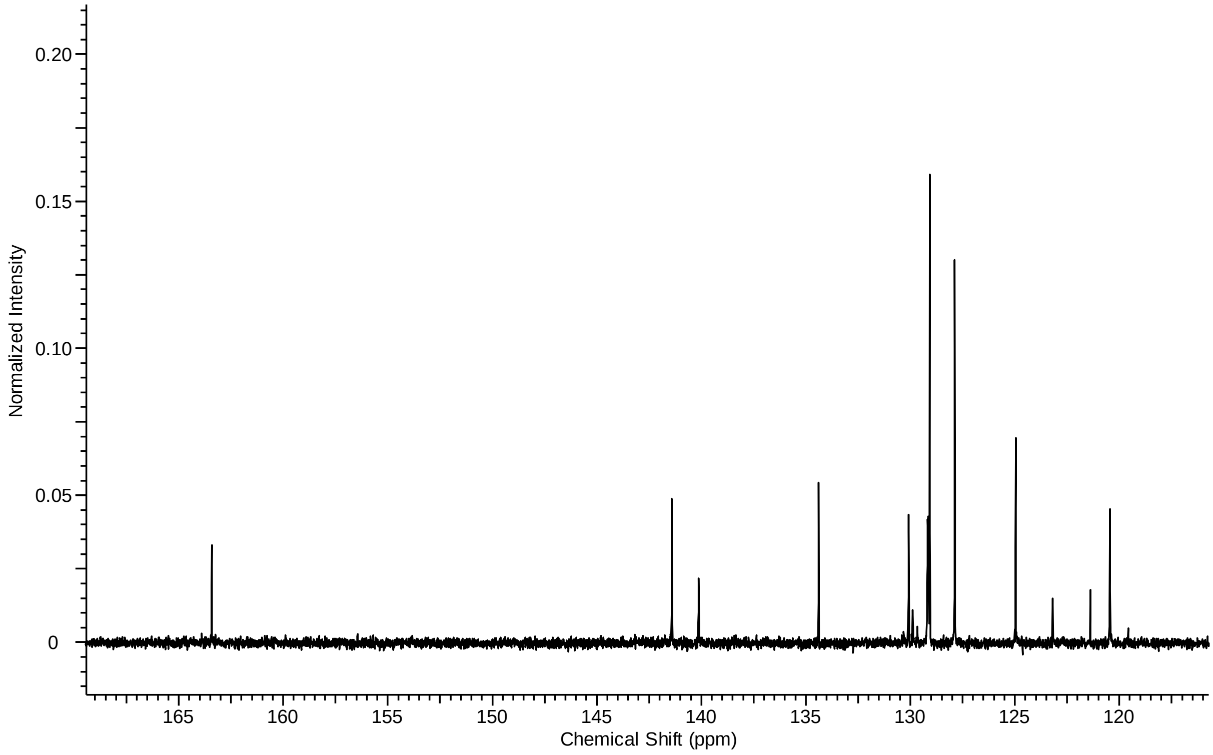

Figure S35. ^{13}C-NMR (DMSO-d_6) spectrum of (2*E*)-*N*-[2,6-dibromo-4-(trifluoromethyl)phenyl]-3-phenylprop-2-enamide (**19**).

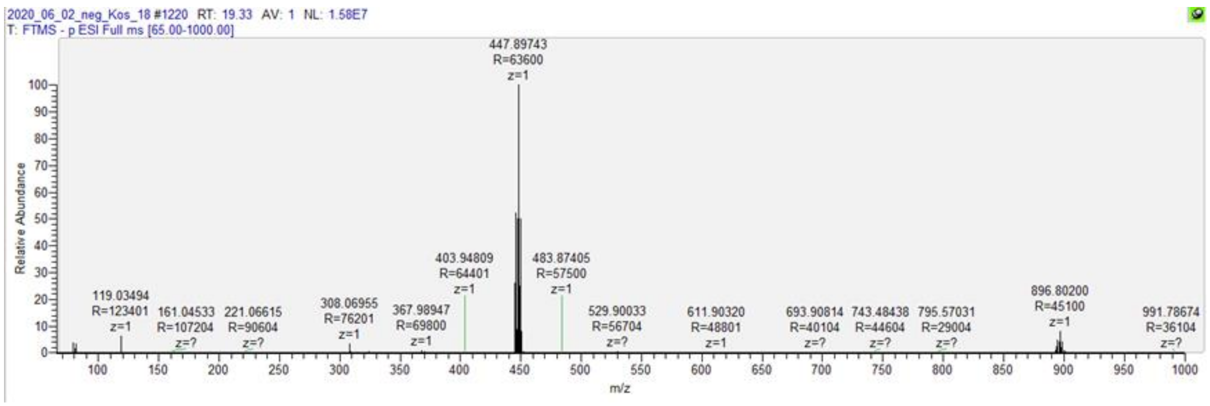

Figure S36. HR-MS record of (2*E*)-*N*-[2,6-dibromo-4-(trifluoromethyl)phenyl]-3-phenylprop-2-enamide (**19**).

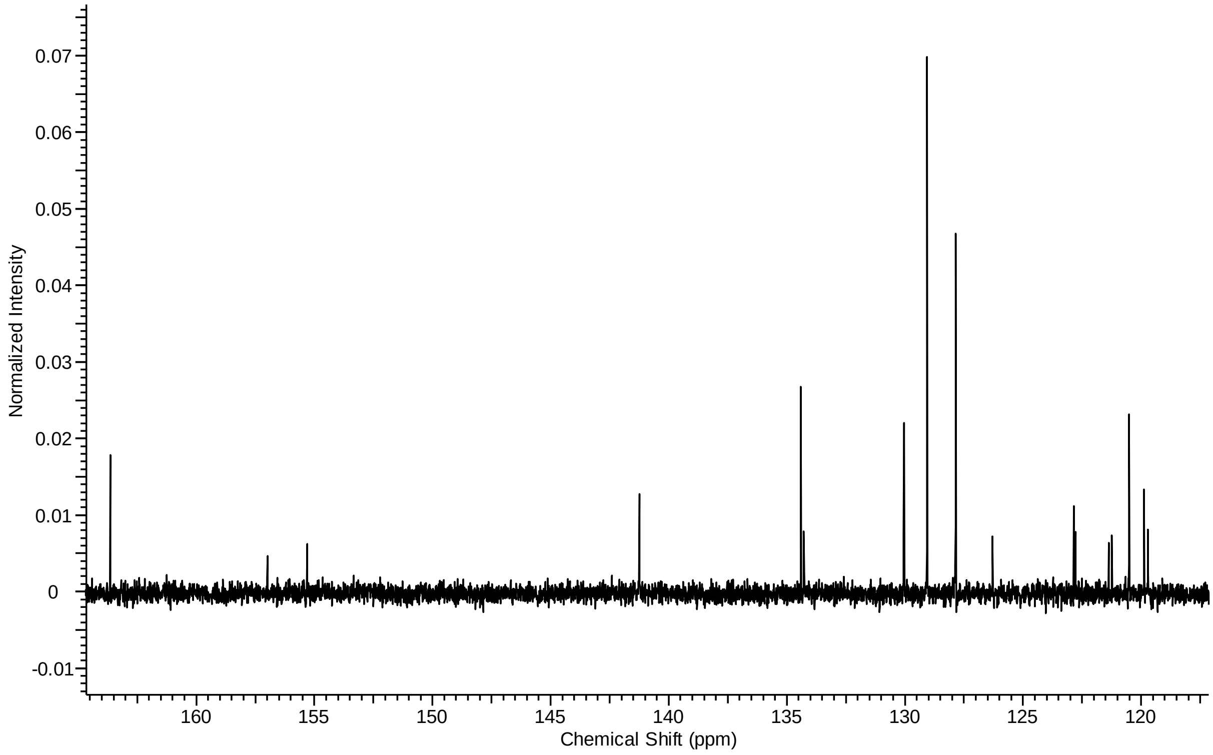

Figure S37. ^{13}C-NMR (DMSO-d_6) spectrum of (2*E*)-*N*-(2,6-dibromo-3-chloro-4-fluorophenyl)-3-phenylprop-2-enamide (**20**).

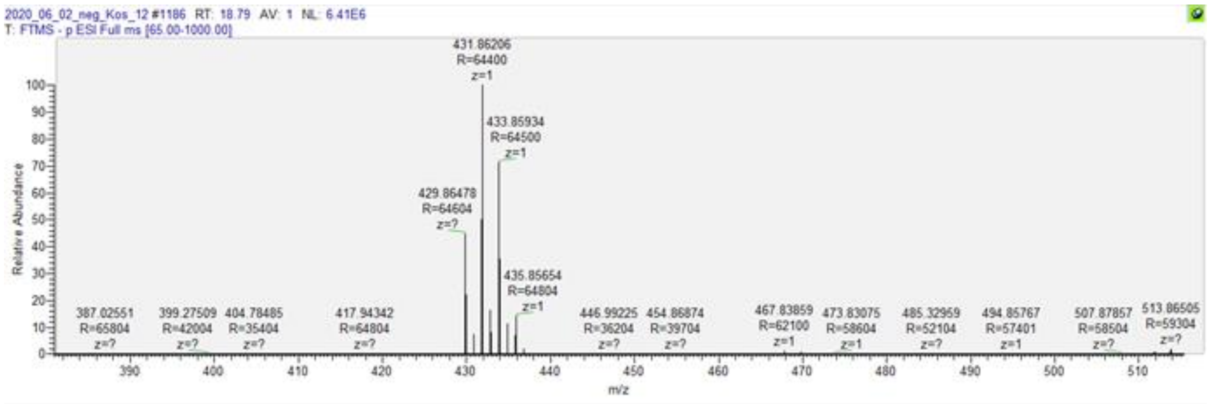

Figure S38. HR-MS record of (2*E*)-*N*-(2,6-dibromo-3-chloro-4-fluorophenyl)-3-phenylprop-2-enamide (**20**).
